# Running-Centred Injury Prevention Support: A Scoping Review on Current Injury Risk Reduction Practices for Runners

**DOI:** 10.1155/tsm2/3007544

**Published:** 2025-02-25

**Authors:** Linda Linton, Jane Culpan, Judith Lane

**Affiliations:** ^1^Dietetics, Nutrition & Biological Sciences, Physiotherapy, Podiatry & Radiography Division, School of Health Sciences, Queen Margaret University, Edinburgh, UK; ^2^Edinburgh Sports Medicine Research Network, Institute for Sport, PE and Health Sciences, University of Edinburgh, Edinburgh, UK; ^3^UK Collaborating Centre on Injury and Illness Prevention in Sport, FASIC Sport and Exercise Medicine Clinic, University of Edinburgh, Edinburgh, UK

**Keywords:** injury, injury prevention, prehabilitation, running-related injury

## Abstract

**Background:** Runners have not benefited from the same reduction in injury rates seen in injury prevention studies conducted in other sports.

**Objectives:** The purpose of this scoping review was to identify and map injury risk reduction practices for running-related injury (RRI), methods of delivery and understand the views of experts and runner's preferences in reducing RRI.

**Design:** Scoping review.

**Methods:** We conducted systematic database searches of MEDLINE, CINAHL and SPORTDiscus from 2000 to April 2024. Eligible studies included injury prevention strategies for RRI. Data synthesis was conducted according to PRISMA Extension for Scoping Reviews using Joanna Briggs Institute framework methodology. Extracted data were mapped and coded from intervention studies, expert opinions and reviews, and inductive thematic analysis created subthemes and themes from prospective cohorts, qualitative studies and surveys.

**Results:** A total of 3777 studies were identified, and 106 studies met the inclusion criteria. In intervention studies, supervision and support appeared critical for better effect. Key injury prevention topics were strengthening, gait re-education and wearables, graduated running programmes, footwear, recovery and educational advice. A multifactorial approach considering individual risk profiles was recommended by experts, but there was a disparity in what runners do to reduce injury risk compared to expert advice, with actions appearing to be related to self-efficacy rather than avoidance.

**Conclusion:** This scoping review highlighted runners require individualized, supported and multifactorial approaches for injury risk reduction, and runners seek knowledge on purpose. We found runners injury risk reduction practices should begin early with youth runners and facilitating this through coaching supervision is likely to support behaviour change. Strategies such as gait retraining, wearables and recovery need further exploration but provide promising strategies runners may engage with more. As runners are likely to choose familiar options minimally impacting lifestyle and running, they should be provided with education but need support with their choices to influence beliefs.

## 1. Introduction

Current injury risk reduction strategies for runners have had a variable impact on reducing overall running-related injuries (RRIs), with the incidence of injuries still higher than in most sports [1]. Risk factors for RRIs include relationships between runner's health, biomechanics, physical conditioning, running environment and training habits, current and previous experience, and behaviours [2]. This range of influences poses difficulties in determining an approach to injury risk reduction practices, where RRIs are predominantly overuse [1, 3], and the aetiology of injury follows a causal framework where loading alone cannot be responsible for injury without considering an interplay of factors such as runner's capacity for load [4, 5]. Additionally, runners have personalized responses to perception of pain, or views on risk which are both dependent on multiple influences characterized by current understanding of biomedical and biopsychosocial models of pain [6, 7] therefore may struggle to interpret and respond to symptoms. Indeed, runners report a range of sensations before classifying themselves as “injured” and describe injury only when they have got to the point of needing a consultation with a healthcare professional (HCP) or cessation of running [8]. This can make it hard for ‘uninjured' runners to engage with injury risk reduction practices. Footballers experiencing discomfort not affecting training were 2.8–5.9 times more likely to sustain a time loss injury the following week [9]. Therefore, having knowledge to intervene prior to this stage and ‘prevent' injury escalating is likely beneficial for injury risk reduction in runners, but very few studies explore runner's behaviours and beliefs [10, 11].

Exercise-based injury prevention programmes (IPPs) in particular have been highly successful across a range of sports and are effective at reducing the risk of injury on average by 29%, but generally not found effective if delivered as an unsupervised intervention [12]. Lauersen, Bertelsen, and Andersen reported a 47% reduction in overuse injury with IPPs implemented across a range of sports, but the overuse injuries reported were predominantly with military participants and no running-specific studies were included in their analysis [13]. Similarly, other studies have found a lack of conclusive evidence for the effectiveness of IPPs designed to address risk factors in runners [14], in endurance runners [15], and for knee injuries [16]. Wu et al. found only three out of nine studies conducted with endurance runners had supervised IPPs, although compliance was higher in these interventions [15]. The majority of supervised IPPs are in team sports [12, 13], where the training environment is supervised by coaches who can influence team participation in IPPs and enhance compliance with injury risk reduction practices [17, 18]. Other factors such as load monitoring can be controlled in team environments through technology such as GPS and coaching plans [19]. Runners participating in differing environments with individual risk profiles, abilities, running goals and beliefs may make universal injury risk reduction interventions more difficult to disseminate and adhere to [20]. Due to these difficulties in supporting exercise-based approaches for runners, running injury prevention research has adopted other approaches such as advice only [21–28], footwear-specific [29–35] and graduated training programmes [36–40]. Despite this growing body of research utilizing strategies *other* than IPPs, there has not been a reduction in RRI like the results seen with IPPs in other sports with over half of runners reporting RRI in a recent survey conducted over an 18-month period in 87 countries [41].

The plethora of different types of injury risk reduction practices coupled with the complexity of RRIs and the diversity of runners [4, 5] provide difficulty in navigating the literature and identifying the direction of future research and dissemination of injury risk reduction practices to runners. Therefore, this scoping review aimed to collate literature on injury prevention strategies used with runners and establish the types of interventions which have been implemented or discussed in the literature to reduce the risk of injury. To provide context to current research, a secondary aim sought to explore how these strategies have been delivered to and received by runners, and what views other stakeholders and experts consider as best practice. The objectives were to review the literature to (i) identify current types of interventions that have been used in the literature for injury risk reduction in runners, what methods have been used to deliver these strategies to runners and what effect have they had (ii) understand experts' knowledge and recommendations on injury risk profiles for reducing risk of RRI, and (iii) explore the behaviours, and beliefs of runners and other stakeholders for what they view to be effective and the factors influencing this. Findings can influence future RRI risk reduction practices for HCPs, runners and other stakeholders.

## 2. Methods

Scoping review methodology was chosen to review current knowledge and practice within this emerging body of literature on injury risk reduction practices and methods of delivery. This provides a broader and deeper understanding not just of current literature from researchers and experts, but the ability to encompass the views of the main stakeholders the runners themselves, running coaches and HCPs, and was conducted according to the Preferred Reporting Items for Systematic Reviews and meta-Analyses extension for Scoping Reviews (PRISMA-ScR) statement [42] and used the Joanna Briggs Institute (JBI) framework methodology for scoping reviews as described by Peters et al. [43]. The scoping review protocol was discussed by all three authors and registered and published on 30th June 2023 with Open Science Framework (OSF) (https://osf.io/2jtqn).

### 2.1. Literature Search Strategy

Prior to the main search and on advice from the University Librarian, a preliminary search was conducted using the database EMBASE to develop keywords and phrases. The final literature search was conducted from inception to July 2023 and then updated from July 2023 to April 2024 through databases MEDLINE, CINAHL and SPORTDiscus. Details of search strategy, subject headings and keywords can be found in Supporting File 1. All citations were uploaded onto the platform Zotero (version 6.0.13), and duplicates were removed. The remaining studies were uploaded to Covidence (https://www.covidence.org/) for screening.

### 2.2. Study Selection

Titles and abstracts were independently screened by two authors (L.L. and J.L.). Criteria for inclusion were set as follows: any type of running, any age, English language, RRI prevention all interventions, RRI prevention all methods of delivery and all stakeholders involved with injury risk reduction in runners such as coaches and HCPs. Literature included could be in the form of studies directly related to RRI prevention approaches. Research studies such as surveys, randomized controlled trials (RCTs), cross-sectional and longitudinal cohort studies and qualitative studies were all considered, alongside reviews, and papers such as reports, commentaries, editorials and opinion pieces. The exclusion criteria included animal and military studies, and reviews which included studies with participant groups other than runners such as the military. Studies focusing only on risk factors that focus on the management of injury rather than injury prevention or epidemiology studies not directly related to injury prevention interventions or prevalence were excluded. A third reviewer (J.C.) was on hand for discussion should any disagreement arise.

On completion of abstract and title screening, full-text studies were retrieved by L.L. and assessed for eligibility by two reviewers (L.L. and J.L.) using the inclusion and exclusion criteria. Reasons for excluding ineligible studies were recorded, and if consensus could not be made at this stage, a third reviewer (J.C.) was available to be consulted.

### 2.3. Data Extraction

A form was developed and customized for the purposes of this study on Covidence to record data extracted from each of the studies. The entities agreed for data extraction were as follows: author and year of publication, type of study or methodology, type of running, study participants' age and sex. For prospective longitudinal and retrospective intervention studies and cross-sectional studies, injury prevention interventions and the type of injury(s) the study aimed to collate, the methods of delivery and efficacy were extracted. For surveys and qualitative studies, reviews, expert opinion papers, editorials, and systematic reviews and meta-analysis, the main themes and outcomes were recorded. All three authors (L.L., J.L. and J.C.) independently extracted all entities for 10 studies to ensure consensus was reached for all information recorded and to assess the practicality of the customized form on Covidence. Following this, two data extractors (L.L. and J.L.) independently extracted data from all remaining studies and met to discuss those studies that Covidence highlighted as having a conflict of agreement. At this stage, reference lists from the current studies were hand-searched for any remaining sources to be added to the data extraction stage.

### 2.4. Data Charting

On completion of data extraction and agreement between extractors L.L. and J.L., all data extracted were uploaded from Covidence to Excel (Microsoft Office Professional Plus 2016) for data synthesis. The primary goal was to analyse studies and chart the data for injury prevention strategies, their methods of delivery and establish views of researchers, runners, HCPs, experts and other stakeholders, and ensure all involved parties' engagement, beliefs and participation and role in injury risk reduction for runners were captured. Due to the diversity in the literature, there is no generic quality assessment tool available that was deemed as applicable, and assessment of quality is not considered essential for a scoping review [43]. However, to provide context to the level of quality of intervention studies, the Physiotherapy Evidence Database (PEDro) scale (https://pedro.org.au/) was used to assess the methodological quality of all RCTs.

Three summary tables were compiled to improve the ease of analysis based on the study design. Each group was analysed separately in order to adequately interpret and map data. Group 1 studies comprised RCTs and were analysed for the type of injury risk reduction strategy used during the intervention, and whether the method of delivery was supervised or unsupervised. Based on the type of intervention and method of delivery, the outcome was summarized as either effective and reduced injury (coded green) or not was not effective at reducing injury (coded red). If the outcome between studies with the same method of delivery of the intervention did not agree between studies (effective and reduced injury/not effective at reducing injury), or the findings reported there was some effect from subgroup analysis, the outcome was coded neutral or undecided (amber). Group 2 studies were narrative reviews, editorials, opinion papers, commentaries, and systematic reviews and meta-analysis and were analysed deductively where initial themes were developed based on the themes or entities which had arisen from Group 1 on interventions recommended for injury prevention. Mapping and grouping these themes guided corresponding attributes or subthemes which emerged from the data and were collated. This approach is described elsewhere by Naem et al., as a ‘top-down approach', guided by existing theories (current interventions) [44]. Group 3 included feasibility studies, cross-sectional and prospective and retrospective longitudinal cohort studies, qualitative studies and surveys. Thematic analysis was used for this group following an inductive approach. Summarized outcomes of studies provided codes which created subthemes, and themes emerged, allowing the runners and other stakeholders a voice and aligning with a constructivist paradigm and therefore deeper exploration of the data [44].

## 3. Results

**Figure 1:**
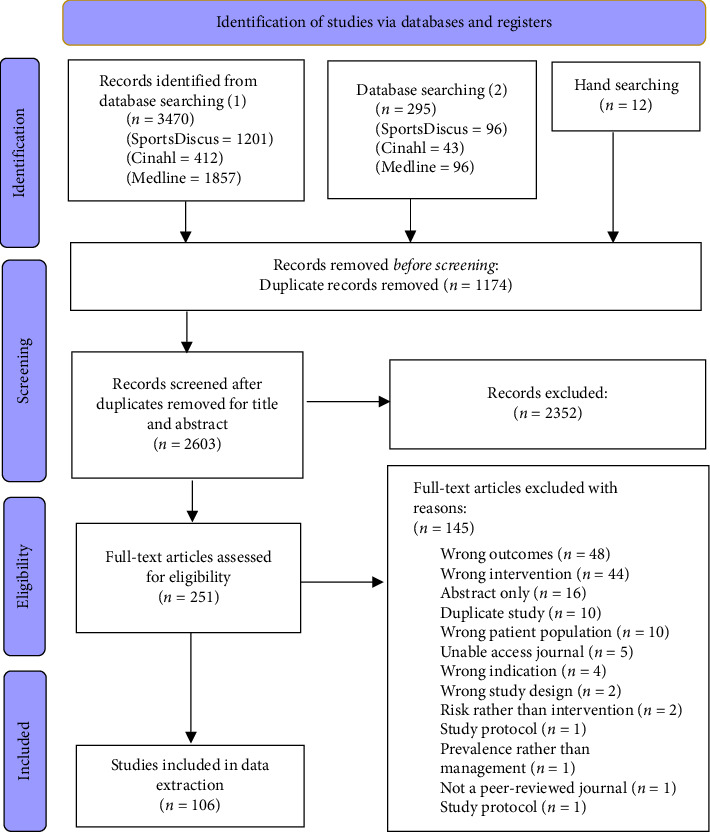
PRISMA flow diagram (1) first database search 2000 to July 2023. (2) second database search from July 2023 to April 2024.

The literature search identified 3470 potential studies from January 2000 to July 2023. After duplicates were removed, 2388 studies underwent title and abstract screening excluding a further 2170 studies, leaving 218 studies to undergo full-text screening. A total of 99 studies were eligible for data extraction which included 12 from hand-searching. The second literature search conducted included 295 studies from July 2023 to April 2024. After duplicates were removed and studies excluded during title, abstract and full-text screening, a further 7 studies were included in the final review. This provided 106 studies in total for analysis.  Figure 1 illustrates an overview of this process and details reasons for full article exclusions. Specific exclusions include abstracts (*n* = 16) published for conferences only, and when unable to access a journal, the corresponding author was contacted via email. If there was no reply following a reminder email, the study was excluded (*n* = 5).

Studies were categorized into three groups for data visualization and analysis and summarized in Tables 1-3 (full details of data extraction can be found in Supporting File 2, and quality assessment for Group 1 studies in Supporting File 3). Overall, intervention studies, reviews and expert opinion papers focused on novice or recreational runners. Elite runners tended to be youth, collegiate or younger adults.

### 3.1. Types of Interventions and Methods of Delivery for Injury Risk Reduction in Runners

**Figure 2:**
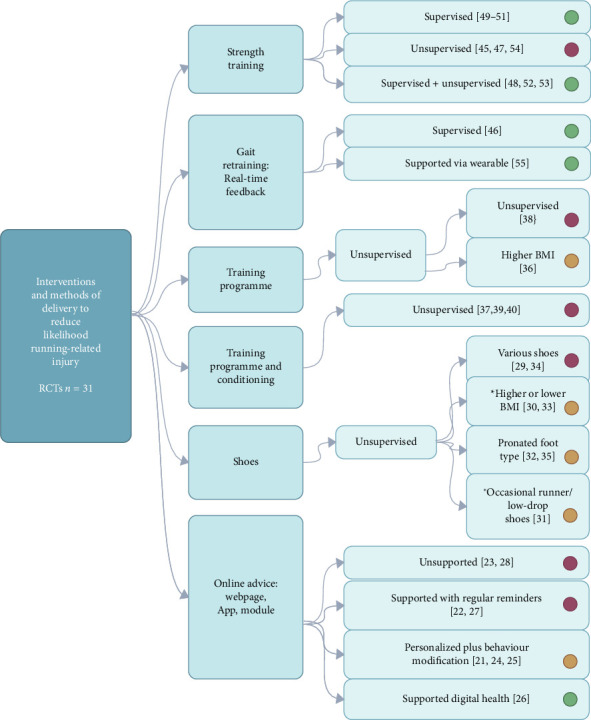
Illustration of randomized controlled trials (RCTs) and types of interventions to reduce risk of injury in runners, and their methods of delivery (supervised, unsupervised, supported or unsupported) with study reference numbers. Green = effective at reducing injury risk, amber = some effect through subgroup analysis or no agreement between studies, red = no effect at reducing injury risk. BMI = body mass index. *n* = number of studies. ⁣^∗^subgroup analysis.

Group 1 had 31 RCTs, with a total of 14,414 participants: 6878 were females (48%), ranging from 22 to 4050. Mean age of overall runners was 35.45 years (age range 12.8–48.2). A variety of techniques were used to distribute information and facilitate interventions throughout each study. In brief, interventions either included some level of supervision or digital support from the project teams, or the participants self-managed with minimal face-to-face interaction or remote support. Each RCT investigating an intervention for injury risk reduction focused on individual injury risk practices. Strength training was delivered as either supervised sessions [48–53] or programmes were provided for participants to perform independently [45, 47, 54]. Gait retraining studies used real-time feedback to facilitate change and were either supervised [46] or provided through digital feedback via wearables [55]. Graduated training programmes were predominantly unsupervised [36–40]. Footwear studies provided shoes for participants, and they trained independently with no further supervision [29–35]. Online interventions varied with some providing access to the website or app that had generic information for the participants to access independently throughout the study [22, 23]. Others provided regular reminders to access the website or app using techniques via email reminder or newsletter [25, 27]. Others used online techniques to establish individual profiles of the runners and tailor their information to their specific needs [21, 24, 27]. Another study provided online modules on digital health for participants to access and, as this was in youth runners, parents were involved in accessing information [26]. Studies collated RRI as all injuries related to running [21, 26–29, 34, 36, 39, 40, 46, 49, 52, 54, 55], injuries of the lower extremity [25, 33, 48, 51], lower extremity and lower back [22, 23, 30, 32, 35, 37, 38, 45, 47, 50, 53] and all injuries related to running, integumentary systems and concussion [24]. Only one study intervention specifically targeted an injury type and anatomical area, and this was ankle injuries [48]. Types of interventions, their methods of delivery and levels of effectiveness are summarized in Table 1 and illustrated in Figure 2. We excluded Kemler, Cornelissen and Gouttebarge from Group 1 analysis as they did not report an injury rate Kemler et al., [122], and details of this study have been summarized in Table 3.

### 3.2. Opinions of Experts, and Reviews and Best Practice for Injury Risk Reduction

**Figure 3:**
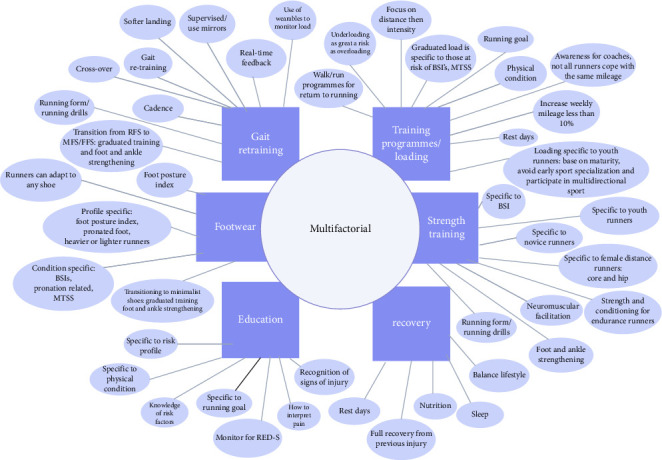
Diagram illustrating themes from expert opinions and reviews on strategies to reduce running-related injury which all interconnect (gait retraining, training programmes and load management, strength training, recovery, education and footwear). Associated attributes or subthemes provide further context to the types of interventions experts see as important or influence injury risk reduction practices. Bone stress injuries (BSI), forefoot strike (FFS), healthcare professionals (HCPs), medial tibial stress syndrome (MTSS), midfoot strike (MFS), rear-foot strike (RFS), relative energy deficiency syndrome (RED-S), running-related injury (RRI).

Strategies described by experts for injury risk reduction were summarized in Group 2 studies, and themes are mapped and coded in Table 2. There were 20 expert view/editorial/opinion papers, seven narrative reviews, five reviews, four systematic reviews with meta-analysis, two commentaries, one Cochrane review and one scoping review. Themes were initially influenced by interventions used in RCTs but closer inspection of Group 2 studies revealed recovery, and education as 2 further entities. Six entities or themes related to injury prevention strategies emerged: gait re-education [5, 16, 56, 57, 60, 63–66, 73, 75–77, 80, 83, 85–89], training/loading [5, 16, 20, 56, 57, 60, 62–64, 67–69, 74–78, 81, 84, 85, 87–89], footwear [16, 56, 60–62, 68–77, 82, 86, 123], strength training [11, 15, 56, 58–64, 66–69, 78–80, 82, 86–88], education [5, 11, 56, 60, 70, 72, 73, 78, 79, 82, 85–88] and recovery [5, 11, 20, 60, 69, 76, 78, 79, 88]. Corresponding attributes or subthemes from each theme detail experts' conclusions and influencers for strategies for the reduction of RRI. Overall, most studies advocated a multifactorial approach of more than one strategy to reduce the likelihood of RRI. The visualization of themes and subthemes is illustrated in Figure 3.

### 3.3. Influencers for Injury Risk Reduction Practice

Group 3 comprised 14 surveys, 10 prospective cohort studies, six qualitative studies, two feasibility studies and one RCT, retrospective cohort study and cross-sectional study. The views and perspectives of runners and a range of key stakeholders are summarized in Table 3. Themes emerged as follows: guidance and supervision, education, technology, beliefs behaviours and self-efficacy, and injury risk profiles. These provide depth to the contextual factors involved in the delivery of injury prevention intervention and specific drivers influencing implementation.

In summary, coaches and HCPs are in the position to provide education to runners [96–103, 122], with guidance and supervision recommended for facilitation [90–95]. Coaches look to HCPs for advice on best practice on injury risk reduction practices [93], and runners look towards HPCs, coaches, running stores or online sources for information [98, 100, 101, 103, 122]. Knowledge on the purpose and benefits of specific injury risk reduction strategies is likely to provide enhanced benefit [95, 97]. Not all runners train with a coach, but welcome a group environment with a professional to seek further education [100]. Despite access to coaches and HCPs, runners exhibit behaviours and beliefs which underpin their choices and veer towards passive, accessible strategies [10, 106, 107, 109–113]. HCPs should respect a runner's autonomy but can support a runner's decision-making [108, 111], and technology could be an area runners may adopt for injury prevention that could provide self-efficacy but needs to provide personalization [104, 105, 108]. As runners have specific unique injury risk profiles, identifying and responding to these when choosing or incorporating IPPs and advice could enhance a runner's response to injury [114–121, 124].

## 4. Discussion

This scoping review found injury prevention approaches for the reduction of RRI have different outcomes dependent on methods of facilitation. Interventions such as strength training are more effective when supervised, and support plus behaviour modification is needed when runners are provided online educational advice. In addition, runners have an individual injury risk profile, and this may be in part why generic, singular IPPs for large cohorts of runners have had limited effect to date. For example, footwear appears to be most effective for specific foot type, and higher or lower BMI, and a graduated training approach was only found beneficial for a specific cohort of runners with higher BMI. Our results found a disparity between current research and the views of experts, with experts favouring a multifactorial approach to injury risk reduction strategies. There is generally more engagement from previously injured runners, and thus, future research must aim to make *all* runners the centre of injury risk reduction practice with their views and preferences helping shape best practice. Promising injury risk reduction strategies recommended by experts such as recovery and gait re-education need more robust investigation, but may be more appealing to runners as they require the least time or effort to adopt and have a limited impact on other parts of daily life.

### 4.1. Runner's Behaviours on Injury Risk Reduction

Supports or shoes are frequently chosen by runners for injury risk reduction [10, 96, 97, 106, 107, 112, 124]. They provide minimal impact on training habits and are dependent on a runner's injury risk profile, and some studies have found shoes effective in reducing injury [30, 33, 35]. Shoe choice is personalized to individual runners, and this demonstrates behaviours of autonomy and motivation for injury prevention as described by Chan et al., which is important for health and well-being [125]. Despite demonstrating self-efficacy with choosing running shoes and high compliance rates in studies in comparison with other injury risk reduction interventions [74], we found runners lacked knowledge or ignored “additional” measures advised when adapting to minimalist shoes [56, 64, 66, 67]. Therefore, runners have confidence in choosing shoes but may not necessarily make time for supplementary actions. A survey on runners and expert's knowledge on running shoes found their views changed after completing an online module [97], highlighting the value of education in ensuring an understanding of best practice on footwear, reasons for change and how to transition safely.

The current scoping review found gait retraining was frequently advocated by expert opinions, reviews and qualitative studies, and runners expressed that this is a strategy they would like to incorporate into their running to reduce the risk of injury [5, 16, 56, 57, 60, 63–66, 73, 75–77, 80, 83, 85–89, 92, 100]. With only two RCTs to date on the effect of gait re-education for the reduction of RRI [46, 55], more research is needed in this area. Easy application of running cues may be especially appealing to novice runners inexperienced in running [126], or developing a comfortable running style similar to running shoe comfort may positively influence engagement with gait re-education techniques [75]. With advances in wearable technology for gait retraining, this digital form of feedback providing runners with motivation and load monitoring may demonstrate improved compliance with interventions in future studies and influence long-term adherence thereafter [105]. However, one study reported wearables increased RRI by inducing competitiveness and influencing poor decision-making, highlighting wearables should also provide personalized support to reduce risk-taking behaviours [104].

### 4.2. The Effect of Supervision and Support for Injury Risk Reduction

Runners are keen to adopt strategies such as shoes, supports and gait re-education which are easy to implement within running training, and also invest extra time on stretching despite a lack of evidence of an effect on injury rate [112, 113, 127, 128]. In contrast to stretching, there is some efficacy for strengthening which runners claim to *know*, but knowledge alone is not enough to influence behaviour [100]. Focusing on strategies to overcome the obstacles such as reminders, monitoring and providing support has been shown to be more effective in influencing behaviours [129]. This concurs with findings from this current study where strength training was only effective as a supervised programme [48–53]. Runners may be open to replace stretching with strengthening if they know why experts recommend this for reducing the risk of RRI [11, 15, 56, 58–64, 66–69, 78–80, 82, 86–88], and have access to educational workshops or runner-specific strength training [10, 100], or can participate in a running group where coaches are able to incorporate strength training into a warm-up [93, 101].

Educating running coaches to utilize coaching skills to influence behavioural change in runners could be beneficial for implementing injury risk reduction practices, especially as runners conversely look to coaches for advice [93, 103]. This has previously been trialled with workshops for coaches in other sports to improve the dissemination of exercise-based IPPs [130]. Coaches are highly influential for athlete's knowledge and beliefs, therefore ensuring that coaches are engaged with the concept of injury prevention and equipped with best practice using a ‘train-the-trainer' approach has been recently highlighted in the literature [131]. Future research should focus on ways of facilitating runners to engage with running groups, an online community, organized workshops or supported digital health modules.

### 4.3. Specific Injury Risk Profiles and Education

In other sports, IPPs have been tailored to target injuries such as hamstring injuries [17], but only one intervention study discussed in this scoping review targeted a specific injury: ankle injuries in orienteers [48]. Runners too have risk profiles for specific injuries such as BSIs [88], but our current study found a lack of research on condition-specific or runner-specific programmes. Youth distance runners are a running cohort where experts endorse specific practices to reduce RRI such as strength training, avoiding early specialization in distance running and ensuring runners perform multidirectional sports [69, 82, 88, 91], particularly in females, who are at higher risk of experiencing changes to bone architecture during youth leading to bone health problems in later life [88, 118, 121, 132, 133]. With only two RCTs in this youth age group, there is scope for further research [26, 51]. One study disseminated a digital health intervention and involving everyone in the youth runner's welfare showed that an approach providing a wider support group was effective in injury risk reduction [26]. This particular study included participants from 12 to 15 years old, and although Tenforde et al. defined the youth runner as any age below 18 years old, most injuries appear to be reported between ages 12 and 14 highlighting specific needs at this age as critical for longevity in running [82]. Similar to youth sport, there is a dearth of RRI risk reduction intervention studies on elite runners [48, 60, 94], possibly reflective of a smaller, younger cohort, with easier access to support staff but potentially different needs. We found experts advocated benefits of recovery in both elite and recreational running such as sleep, balancing lifestyle, rest days, nutrition and ensuring full recovery from injury [5, 11, 20, 60, 69, 76, 78, 79, 88], but it is not clear from runners how often they utilize such practices, especially as these often occur out with actual running training and future studies should try to capture this information. As there are complex reasons, runners engage with running [134], providing runners flexibility to adapt training and recovery within daily life and gaining understanding of runner's viewpoints may improve compliance [134].

There is minimal research on the effects of educating runners on injury definition despite studies in this scoping review reporting this is important [5, 11, 56, 70, 79, 85, 87, 88]. As identified in this scoping review (Table 1), studies investigating interventions to reduce the likelihood of RRI had different criteria on defining RRI location, and therefore, agreement is needed between researchers on what is classified as an RRI. Runners in turn have a different understanding of when they should respond to pain based on their interpretation that they are only injured if needing to seek professional help or are unable to run [8]. Yet, intervening prior to the cessation of running may *prevent* further injury. Education on pain monitoring tools for adapting training loads in response to pain has been suggested in the literature as a strategy to reduce likelihood of RRI [93, 135, 136]. It appears experienced runners have more strategies than novice runners to adapt and flex their training programme in response to musculoskeletal pain [137], which novice runners lack awareness of [115, 119, 138]. Interestingly, graduated running programmes on their own have had little traction in reducing RRI risk for runners new to running, with no clear benefit for shorter or longer walk-run training programmes [36, 38, 115, 119], or with additional conditioning [37, 39, 40]. With new runners having different responses to loading dependent on body weight, age, running pace and previous injury [36, 37, 40, 114, 115, 119], this highlights the need for further research to investigate the effect of a multifactorial approach involving personalized running programmes and education.

Researchers have shown promising developments with online tailored programmes, but only if individualized, and provide reminders. The use of outcome measures for behavioural modification in response to educational interventions appears promising [11, 21, 24, 28]. Runners rarely access content unless injured [23, 25] and generally show more interest and investment in injury reduction practices than noninjured runners, reflecting their injury never fully resolved and they seek prehabilitation as a means for rehabilitation [100, 107]. As the previous injury is a strong predictor for future injury and with approximately 50% of runners currently running with some elements of discomfort [41, 139], giving runners support with strategies that are evidence-based and easily incorporated into usual running training will help aid longevity with running. Therefore, *all* determinants and injury risk profiles need to be considered when engaging in a new activity particularly running, with appropriate supported education given *alongside* a graduated running programme.

### 4.4. Limitations

This scoping review is one of the first to consider the wide range of injury prevention strategies available to runners, coaches and HCPs giving deeper insight into the multifactorial nature of injury prevention. Data collected within this scoping review should be taken in the context of the broad range of types of studies identified and accessed. We did not consider studies prior to 2000, and this was based on the majority of studies before this having been conducted on military participants which were considered to have variables other than running to have an effect on injury and types of injury, hence the exclusion of Pope et al. and Yeung et al. [140, 141]. Methods used in the included studies were heterogeneous, but we believe this has provided a broad range of diverse views, and the inclusion of surveys and qualitative studies added a deeper more meaningful significance to experts' viewpoints and findings from quantitative studies. A number of reviews summarized in Table 2 and illustrated in Figure 3 may have included the same original research studies, and thus, the conclusions drawn from this may be over-represented. In view of this, weighting was not applied to each specific intervention highlighted in Table 2.

Epidemiology studies were excluded as although these provide associations and risk factors and hypotheses on what could or should prevent injury, they were not included unless they looked specifically at interventions and associations with preventing RRI. Studies directly investigating risk factors for RRI were also excluded where possible to avoid making assumptions based solely on risk factors and to ensure the relevant literature discussing proactive strategies to reduce the likelihood of injury was highlighted. The average age of runners in this current scoping review including novice runners appears higher than other studies conducted in different sports on injury risk reduction practices [12], and this may need to be investigated in future studies to identify whether there are specific risk profiles for people taking up running later in life, perhaps following a previously sedentary lifestyle.

## 5. Conclusion

This scoping review identified strength training, gait re-education, graduated running programmes, footwear and online education as the main strategies investigated in intervention studies on injury risk reduction practices in runners. Additionally, recovery strategies have been recommended by experts, but no research studies have yet investigated this. Current practices reducing the risk of injury have been largely unsuccessful, not because they have not addressed risk factors, but possibly due to the method of delivery. It appears key that where more supervision and support have been provided alongside interventions, the outcome for reducing RRI is more effective. Also, in contrast to the linear approach to injury risk reduction practices in research studies, experts believe a multifactorial approach as most beneficial. Through this approach, runners may be provided and supported with the capacity to make informed decisions when adopting injury risk reduction strategies, influence goal setting and learn more about their own individualized injury risk profile. If this entails runners choosing the most convenient options or strategies that enforce a sense of self-efficacy, then we should ensure they have knowledge for best practice. Further research on runner's preferences and areas such as recovery and wearables, and specific risk profiles such as youth or interventions for specific injuries is needed ensuring that the runners' views and needs are at the centre of the development of future injury risk reduction research and that changes in behaviour modification are just as an important an outcome measure as a reduction in injury. Having support from peers within a group environment, supervision from HCPs and coaches, using wearables with personalized feedback or developing educational digital health is likely the most effective means for facilitating injury risk reduction practices in runners.

## 6. Perspectives and Recommendations for Future Research

Runners are interested in learning about injury prevention approaches, particularly following previous injury, but runners should be given choice, and approaches must be easily incorporated into usual training habits. Promising strategies such as gait re-education, use of wearables and recovery that runners can adopt with little impact on usual running habits need further research to demonstrate efficacy. Research should use outcome measures such as effect on behaviour change as well as reduction in injury to understand runner's response to injury prevention interventions better. Educational personalized online modules addressing runners' individualized injury risk profiles and digital health need further exploration with different populations of runners; however, coaches may be best placed to provide a supervised approach if they have the knowledge. A train-the-trainer approach could provide scope to reach the wider running community. We found experts propose a range of injury risk reduction strategies for runners to address the multifactorial nature of RRI, but current injury prevention RCTs tend to focus on specific risk factors or do not provide supervision for interventions which may explain in part that RRIs remain high in comparison with other sports. There is heterogeneity across the studies selected in this scoping review, which includes all types of running and a range of methodologies. Researchers and HCPs can use the findings to develop more robust research and to help runners engage with strategies to reduce their risk of RRI.

## Figures and Tables

**Table 1:** Group 1 studies comprising randomized controlled trials investigating injury risk reduction in runners.

Author year	Population (n)	Age (years)⁣^∗^	Injury prevention intervention	Comparator	Method of delivery	Injury(s) collated	Outcome	Code
Adriaensens et al. 2014 [21]	Recreational *n* = 214	23.9 (4.3)	Website. Online module to influence injury preventative behaviours	Read magazines	Unsupported	All injuries related to running.	Effective for knowledge, risk behaviour, attitude, intention and injury prevention behaviour at Time Point 1, but less effective Time Point 2/3	A

Baltich et al. 2016 [45]	Novice and recreational *n* = 129	32.8	Group 1. Exercise: Resistance training Group 2. Exercise: Functional strength	Stretching	Unsupervised	Lower extremity or lower back	No difference between groups	R

Bertelsen et al. 2018 [36]	Novice obese *n* = 56	39.2 (9.5)	Graduated running programme starting 3 km/week	Graduated running programme starting 6 km/week	Unsupervised	All muscle, joint, tendons and/or bone injuries	Reduced risk of injury for more graduated running programme	A

Bredeweg et al. 2012 [37]	Novice *n* = 432	38.1 (10.8)	Preconditioning plus graduated running programme	Graduated running programme (no preconditioning)	Unsupervised	Lower extremity or lower back	No difference between groups	R

Buist et al. 2008 [38]	Novice *n* = 532	39.8 (10.1)	Graduated running programme	Standard running programme	Unsupervised	Lower extremity or lower back	No difference between groups	R

Chan et al. 2018 [46]	Recreational *n* = 320	33.9 (9.5)	Gait retraining with biofeedback	Running, no feedback	Two weeks of supervision	Any running-related musculoskeletal complaint	Reduced risk of injury 62% for gait retraining	G

Cloostermann et al. 2020 [22]	Recreational *n* = 4050	42.3 (12.1)	Online injury prevention programme	Continued usual running preparation for race	Supported fortnightly	Lower extremity or lower back	No difference between groups	R

Desai et al. 2022 [47]	Recreational *n* = 433	39 (8.6)	Exercise: Strength training	Continued usual training	Unsupervised	Lower extremity or lower back	No difference between groups	R

Dubois et al. 2015 [29]	Recreational *n* = 24	31.7 (8.2)	Traditional shoes	Minimalist shoes	Unsupervised	Running-related injury	No difference between groups	R

Fokkema et al. 2019 [23]	Recreational *n* = 2378	41.2 (11.9)	Online injury prevention programme	Continue usual running training	Unsupervised	Lower extremity or lower back	No difference between groups	R

Fuller et al. 2017 [30]	Recreational *n* = 61	27 (7)	Conventional shoes	Minimalist shoes	Unsupervised	Pain in foot, ankle, calf, shin, knee, thigh and lower back	No difference between groups. Subgroup analysis: Increased risk of heavier, wearing minimalist shoes	A

Halvarsson and von Rosen 2019 [48]	Elite orienteers *n* = 62	24.1 (3.6)	Exercise: Neuromuscular control	No input	One supervised, then unsupervised	Lower leg injuries and specifically ankle sprains	No difference between groups. Reduced injury risk when completing exercises more than twice a week	G

Hespanhol, van Mechelen, and Verhagen 2018 [24]	Trail runners *n* = 232	44.5 (9.5)	Online tailored injury prevention advice	Online general advice	Supported fortnightly contact	All musculoskeletal injuries, integumentary systems (blisters, nails) and concussion	No difference between groups, but reduced risk of injury after 6 months.	A

Hollman et al. 2019 [25]	Recreational *n* = 51	48.2	Online tailored injury prevention advice	Online general advice	Supported fortnightly contact	Lower limb	No difference between groups. Positive change is seen towards preventative behaviours.	R

Jacobsson et al. 2023 [26]	Youth track and field *n* = 142	12.8 (2.4)	Online. Digital health platform.	Access to digital health platform after 16 weeks	Supported through emails	Lower limb, upper limb, spine, head and face	Reduced injury risk	G

Letafatkar et al. 2019 [49]	Novice *n* = 49	32.7 (9.0)	Exercise—conditioning training. Exercise—conditioning training/feedback	Trunk and upper limb exercises	Supervised	All musculoskeletal injury	Both conditioning and conditioning plus feedback reduced injury risk	G

Letafarkar, Rabiei and Afshari 2020 [50]	Novice *n* = 60	32.9 (6.1)	Exercise—neuromuscular training Exercise—neuromuscular training plus knee valgus control	Trunk and upper limb exercises	Supervised	Lower extremity or lower back	Both neuromuscular exercise and neuromuscular exercise plus knee valgus control reduced injury risk	G

Lundstrom et al. 2019 [39]	Marathon *n* = 34	20.7 (1.3)	Marathon plan plus core exercises. Marathon plan plus plyometric exercises	Marathon plan	Long-run and high-intensity running supervised	Injury and pain	No difference between groups	R

Malisoux et al. 2016 [31]	Recreational *n* = 553	38.3 (9.7)	Heel-to-toe drop 10 mm Heel-to-toe drop 6 mm Heel-to-toe drop 0 mm		Unsupervised	Lower extremity or lower back	No significant difference between groups. Secondary analysis found lower injury rate in runners using lower drop shoes.	A

Malisoux et al. 2016 [32]	Recreational *n* = 372	40.5 (10)	Standard heel-to-toe drop 10 mm Motion control heel-to-toe drop 10 mm	Lower extremity or lower back	Unsupervised	Lower extremity or lower back	Reduced risk of injury for motion control shoes	G

Malisoux et al. 2019 [33]	Recreational *n* = 848	40.5 (10)	Soft cushioned shoes Hard shoes		Unsupervised	Musculoskeletal injury of the lower limbs	No significant difference between groups. Subgroup analysis: Lighter runners had higher risk of injury	A

Mendez-Rebolledo et al. 2012 [51]	Youth track and field *n* = 22	15.15 (2.4)	Exercise—neuromuscular exercise programme	Continued normal preseason programme	Supervised	Thigh muscle strains, knee bursitis, knee tendinopathy and stress fractures	Reduced risk of injury	G

Ramskov et al. 2018 [40]	Recreational *n* = 447	39.5 (9.9)	Preconditioning 8 weeks of both groups. Intensity and volume progression		Unsupervised	Injury of muscles, tendons, joints or bones	No difference between groups	R

Suda et al. 2022 [52]	Recreational *n* = 118	41 (7.35)	Exercise—foot core training	Static stretching	Supervised weekly for 8 weeks, then unsupervised	Any musculoskeletal injury or pain	Reduced risk of injury. Older age and higher training volume were at higher risk of injury	G

Taddei et al. 2020 [53]	Recreational *n* = 118	40.9 (7.35)	Exercise—foot core training	Static stretching	Supervised weekly for 8 weeks, then unsupervised	Lower extremity or lower back	Reduced risk of injury	G

Thiesen et al. 2014 [34]	Recreational *n* = 247	41.8 (10.4)	Standard running shoes with soft midsole standard running shoes with hard midsole		Unsupervised	Lower limb, upper limb, spine and head	No difference between groups	R

Toresdahl et al. 2020 [54]	Novice marathon *n* = 720	35.9 (9.4)	Exercise—strength training	No intervention	Unsupervised	All injuries	No difference between groups	R

Van Der Does, Kemler and Gouttebarge 2023 [27]	Novice *n* = 741		Online injury prevention with fortnightly emails or accessed once	Continue normal running	Unsupervised but Group 1 received fortnightly emails	Any physical complaint	No difference between groups	R

Van Hooren, Plasqui and Meijer 2024 [55]	Recreational *n* = 172	40.1 (10.8)	Pressure sensors providing real-time feedback with instructions on change	Pressure sensors providing real-time feedback with no instructions on change	Supported through app	Running-related injury	Reduced risk of injury	G

van Iperen et al. 2022 [28]	Long-distance runners *n* = 425	44.65 (11.65)	Online personalized running app	Continue normal training	Unsupervised	Injury or bodily damage	No difference between groups	R

Willems et al. 2021 [35]	Recreational *n* = 372	40.4 (10.5)	Standard neutral shoes Motion control shoes		Unsupervised	Lower extremity or lower back	No difference between groups. Subgroup analysis reduced injury risk for pronation-related RRI if wearing motion control shoes	A

*Note:* Age is reported as mean. Intervention effective in reducing injuries “green” (G); intervention not always effective or only effective with subgroup analysis ‘amber' (A); intervention not effective “red” (R).

⁣^∗^standard deviation.

**Table 2:** Summary of Group 2 studies, opinion and expert view papers, narrative reviews, and systematic reviews and meta-analysis on injury risk reduction in runners.

Author	Type of study	Population	Main aims and objectives	Themes, synthesized themes and frameworks	Codes
Andreyo, Unverzagt, and Schoenfeld 2022 [56]	Review	Not specified	Provide recommendations on who is suitable for this footwear and how to transition to minimalist footwear without injury	Progressive loading, gait retraining, strengthening and adaptation period needed. Runners with higher BMI, females, history stress fractures and previous injury have increased risk of injury. Previous hip, knee and shin injuries reduced risk in minimalist footwear. HCPs help support informed decisions.	G S T F E

Alexander et al. 2022 [16]	Systematic review meta-analysis	Novice and recreational	Injury prevention and management of knee injuries in runners and evaluation of effectiveness.	Reduced risk of knee injury for running technique re-training, footwear. Online injury prevention programmes have little effect on knee injury risk, and multicomponent exercise, graduated running programme, prevention education programmes do not reduce knee injury risk.	G T F

Barton et al. 2016 [57]	Mixed methods	Not specified. All runners	Gait retraining interventions and effect on biomechanical changes, management and injury prevention RRI.	Experts thought gait retraining played a role in injury prevention. Reducing vertical loading rate, higher cadence and forefoot strike/midfoot strike was considered for reduced injury risk.	G T

Blagrove et al. 2020 [58]	Expert view/opinion paper	Adolescent endurance runners	Efficacy of strength training on determinants of endurance running in adolescent runners, and best practice to improve performance and minimize occurrence of overuse injury.	Strength training twice per week that includes resistance and plyometric training, and sprinting is likely to provide benefits to improved performance. Movement skills training and specific strengthening of tissues vulnerable to injury are important for lowering the risk of overuse injury.	S

Brumitt 2009 [59]	Opinion paper/expert view	Cross-country runners	A review on risk factors for RRI and strategies to address these include strengthening core and hip muscles to reduce the risk of injury in female cross-country athletes.	Female cross-country athletes have a greater risk of injury than male. Injury prevention programs for the female cross-country athlete, including core and hip exercises, may reduce the risk of injury	S

Byrne 2014 [60]	Editorial/expert opinion	Cross-country, track and field	An interview with a coach for University students gaining perspective on injury prevention from previous and current experience.	Recovery after sessions, alternate exercise and balancing lifestyle (sleep, study, prehabilitation and nutrition). Individualize training volume. Barefoot running used as foot strengthening. Running form improves efficiency and reduces injury. Strengthen, but coaches need awareness of overload. Medical community to be honest about uncertainty.	R T S G E F

Jaén-Carrillo et al. 2021 [61]	Narrative review	All runners	Review on musculotendinous stiffness in the lower limb used for optimizing performance and reducing injury in runners.	Coaches to adopt specific strengthening incorporating eccentric, isometric and plyometric to increases muscle stiffness, and target stretch shortening cycle. Foot strengthening stiffens foot/arch.	S

Corrarino 2012 [20]	Narrative review	Recreational	Discussion pathology, stress fracture risk fractures and strategies for prevention.	Training surfaces, shoes, shock-absorbing orthoses, decreasing speed, strengthening programmes, graduated training and optimize nutrition for recovery. Graduated training post-BSI.	T F S R

Craig 2008 [62]	Commentary	Recreational	Injury prevention methods most effective in reducing medial tibial stress syndrome in physically active people	Shock-absorbent insoles, age of running shoes, control of pronation, graduated running programmes, improve soleus strength for injury prevention.	F T S

Davis and Futrell 2016 [63]	Narrative review	Not stated	Runners have a threshold for injury which depends on many risk factors: Mechanical structure and load tolerance	Strengthening requires neuromuscular facilitation to aid transition to function. Use real-time feedback during gait retraining and transition from rear to forefoot strike with increased cadence.	S G T

Davis, Rice, and Wearing 2017 [64]	Review	Not stated	Current high rate of RRI and association with changes in foot strike and footwear.	Gradual transitioning when changing to barefoot running, or forefoot strike to reduce injury. Strengthening recommended alongside this to address calf and foot muscles.	T S G

Doyle et al. 2022 [65]	Systematic review meta-analysis	Not specified	This systematic review and meta-analysis looks at RCTs that have used gait retraining and its effect on kinematics, kinetics, pain, performance and injury	Gait retraining effective for injury prevention, in particular reducing landing impact in healthy runners.	G

Hamill and Gruber 2017 [66]	Review	Not specified	Changing foot strike to forefoot strike or midfoot strike and its effect on running economy and reduction of RRI.	A change from rear foot strike to forefoot/midfoot strike alongside strengthening is associated with reduced risk RRI, but is likely to be least beneficial in recreational runners.	G S

Hart and Smith 2013 [67]	Expert view	Not specified	To describe the principles of barefoot running for reduction RRI.	A graduated programme recommended as well as advice to reduce shoe support gradually. No equipment needed. If the barefoot practice is not continued the strengthening effects diminish.	S T

Kemler, Valkenberg and Gouttebarge 2019 [11]	Review plus expert meetings and survey	Novice	3 stages developing intervention promoting behaviour change reduction RRI (included ski injuries): (1) identify problem and economic burden; (2) identify interventions, whom and how should apply these; (3) development of Runfitcheck	Information should be easily accessible and simple, and training schedule, strength training and warm-up specific to physical condition of the runner. Accessible by smartphone/tablet/PC. The main behavioural themes developed were knowledge, awareness and self-efficacy.	E S R

Krabek, Waite and Lipman 2013 [68]	Opinion paper/review	Ultramarathon	To review the literature for preventative strategies in ultramarathon runners for managing injury and illness. Combination of literature and expert opinion.	For injury risk reduction, limit weekly mileage to < 64 km/wk; limit competitions; gradual increase mileage during training 10%–20%; train on same surface as competing on; strengthen hip and knee muscles; have appropriate comfortable gear, test before race. Prevent blisters with foot/shoe care.	T S F

Krabek et al. 2019 [69]	Review	Youth distance runners	Factors related to injury in youth distance runners, with recommendations for injury prevention.	“Readiness of running list”: (1) training programmes on maturity rather than chronological age (2) screen for previous injuries and address strength and neuromuscular training, multidirectional and impact activities (3) avoid early sports specialization (4) rest day/week (5) light trainers (6) multi surfaces (7) adequate calories (8) only compete if following acceptable training programme.	T S R F

Johnson et al. 2023 [70]	Opinion paper	Novice and recreational	Expert opinion on importance of addressing risk factors for RRIs	Complexity of RRI, as injury risk profiles are individual. Shoes benefit specific foot types and strength. Most effective method reducing RRI risk is educating runners to recognize signs of injury earlier.	F E

Mai et al. 2023 [71]	Scoping review	Not specified	A scoping review to look at the design features of running shoes and how individualization of footwear can reduce RRI based on biomechanical risk factors	They found footwear design features related to increasing the risk for one injury related to a biomechanical risk factor, but decrease risk of injury for another biomechanical risk factor for another injury. Footwear needs to be individualized and specific to injury risk profiles.	F

Malisoux and Theisen 2020 [72]	Narrative review	Recreational	The aim of this literature review is to provide clinicians and coaches with evidence-based information on choice of shoes to reduce RRI that they can advise their runners with.	Subgroup runners benefit from different shoe types (1) standard shoes provide a minimum motion control, (2) cushioning for lighter runners, (3) comfort, (4) alternate shoes, (6) transition slowly, (7) runners adapt to shoe type, (8) role of shoe in injury prevention is more complex than the shoe alone.	F E

Murphy, Curry and Matzkin 2013 [73]	Review	Endurance	Review of the literature on the subjects of kinematics, minimalist shoes versus cushioned shoes and barefoot running.	No prediction how runners respond changing from shod to barefoot as injuries are multifactorial, but transition gradually is important. If runner is not presently injured, there is no evidence that changing to barefoot running will prevent them getting an injury.	F E G

Napier and Willy 2021 [5]	Opinion paper	No specified	A causal framework presented which encourages injury prevention interventions to consider injury won't occur without multiple related factors.	Advice: Training programmes have often focused on training volume but should acknowledge a change in load or no change in volume as risk factor, use wearables to monitor. Tailor programme individuals' risk profile: Physical, training, psychological, masters, adolescent, previous injury and RED-S.	T E G R

Nielson et al. 2020 [74]	Educational review	Combination	A rev of RCTs on running injury prevention research and compliance with different interventions. Review of data from 7 RCTs and 1 experimental study on injury prevention and compliance as defined as having completed 90% of the intervention given or if referring to running shoes, having worn them for all running sessions.	Four running programme interventions: At the end of the follow-up period RUNCLEVER 0% compliance [40], PR21 0% [116], Start to Run 21% [114] and GRONORUN 1% [38]. Four running shoe interventions: Theisen et al. [34] 68%, Malisoux et al. [31] 76%, Malisoux et al. [32] 90% and Malisoux et al. [33] 86%. Runners find it easier to wear a specific shoe than to be asked to change or adopt a specific running programme. Experienced recreational runners less inclined to change running practice than novices.	T F

Nigg, Mohr, and Tilp 2019 [75]	Editorial commentary	All	Series of 6 peer commentaries discussing the main article (muscle tuning and preferred movement path—a paradigm shift. Nigg et al. 2017) which discusses 2 paradigms “impact force” and “pronation control” which they propose should not be used in the context of injury prevention for runners and instead replaced with the runners “preferred movement path”. That is, kinematics only change minimally to a change in show and/or orthotics and that it is the runner in the shoe that provides the change.	(1) Further research should reconsider long held beliefs on impact forces, pronation and RRI. (2) There is limited evidence in quantifying impact force with injury. (3) More research is needed on orthotic interventions to see if they change kinematics (4) A paradigm shift is necessary to relate running injury risk, and footwear design with large scale studies are needed. (5) Impact force and ankle pronation do not provide evidence for injury preventative measures in runners, yet other measures have similarly provided little evidence to date. This suggests tissue loading and adaptation responds to stimulus (e.g., footwear, gait changes, and training errors), and all should continue to be explored. (6) There is little evidence supporting paradigms of muscle tuning and preferred movement path, and future research is needed.	F T G

Ramsey 2016 [76]	Opinion paper	Track/cross-country	Paper aimed to assist coaches, trainers and athletes in track/cross-country running to prevent and manage iliotibial injury.	Novice runners most affected. Coaches to monitor training, loading and gait re-education mid- to forefoot strike. Monitor age training shoe age, avoid downhill. Stretching and foam roller suggested.	T G F R

Relph 2022 [72]	Cochrane review	All types of adult runners	To assess the literature on how different characteristics of running shoes can prevent injury	Low to very low certainty evidence: No difference with neutral, cushioned or minimalist shoes for RRI risk in recreational runners; uncertainty motion control shoes v neutral cushioned reduce RRI; soft or hard midsole may reduce RRI; shoes based on foot posture index may reduce RRI.	F

Rixe, Gallo and Silvis 2012 [77]	Opinion paper	Track/cross-country	Review to look at the features of barefoot running and minimalist footwear and their influence on the prevention of RRI	Evidence shows minimalist running style can effect biomechanics, kinetics and change foot strike, but does not reduce RRIs, but simply changes the type of injury. Slow transition to minimalist needed.	F G T

Silva, Ready and Etzel 2020 [78]	Opinion paper	Not specified	This paper discusses a holistic approach to injury risk reduction with increasing performance.	The key themes: Sleep for recovery and performance; rest days; active recovery/cross-training days, especially youth and older runners; nutrition and hydration; mental and emotional health awareness needed coaches and HCPs; dynamic warm-up and cooldowns; lack of strength is a modifiable and can be addressed; ensure adequate recovery from previous injury.	R S E T

Stenerson and Melton 2021 [79]	Opinion paper	Recreational	This paper provides a multifaceted approach to reduction RRI, which involves a combination of education, and balance and hip strengthening exercises.	Multifaceted approach needed to address multiple risk factors. Limited effectiveness of previous injury prevention programmes when the focus is on one risk factor. HCPs and fitness professionals are best placed to provide education to runners, as runners report the barrier is not knowing what to do. Poor balance relates to falls, reduced performance and poor postural control. Single leg, hip abductor exercises incorporated into in-season programmes and resistance training off-season. Dynamic warm-ups.	E S R

Šuc et al. 2022 [80]	Narrative review	Endurance runners	To look at resistance training in endurance athletes and effect on running economy, running biomechanics and RRI, to inform coaches and clinicians programmes.	Improvement running economy, performance and adaptation of neuromuscular system. Plyometric and isometric training improved running economy through reactive strength and tendon stiffness. Ensure foot and ankle strengthening and running technique to reduce RRI risk.	S G

Tenforde et al. 2015 [81]	Opinion paper	Youth athletes	Literature on bone health comparing participation in ball sports, and participation in running in youth athletes.	Peak bone mass occurs during childhood/adolescence. Ball sport athletes have greater bone mineral density than distance runners and therefore should be incorporated in training as prehabilitation.	T

Tenforde et al. 2020 [82]	Expert opinion paper	Youth running	This paper aims to provide recommendations for reducing risk of injury in youth runners.	Participate in higher impact, multidirectional sports during childhood. Early specialization discouraged. Screen for low energy availability. Monitor training loads during growth spurt and compete with maturity rather than chronological age. Perform foot and ankle strengthening exercises, and transitioning from cushioned to minimalist footwear gradually with strengthening.	T E S F

Teyhen 2015 [83]	Expert view	Combination	Summary of a study using real-time feedback for running mechanics to reduce risk of injury	Using visual feedback (mirror) can be useful to improve form when cued/supervised by physical therapists. Audio feedback via the use of a metronome can be used to improve cadence.	G

Teyhen 2014 [84]	Expert view	Combination	Summary of study by Ref. [109]. Advice for people starting running or increasing mileage without injury.	Increasing weekly mileage by over 30% will increase risk of injury, and by less than 10% can reduce risk of injury. Speed plus distance may make runners more susceptible to overuse injuries.	T

Verhagen 2012 [85]	Editorial	Novice	Discussion of health benefits of running, versus impact of injury in novice runners, injury prevention strategies for engagement.	Lack of studies on RRI prevention particularly the novice runner who may have specific injury risk profile. Research needed to assess overloading tissues and underloading. Overweight novice runners higher risk. Mechanics change with fatigue and research needed on kinematics and RRI prevention.	T G E

Vincent and Vincent 2018 [86]	Clinical commentary	Not specified	The aim of this paper is to address focused training of the foot intrinsic muscles for stability and injury prevention.	Authors liken the foot intrinsic muscles to trunk core, providing stability during single-leg activation activities in running. Poor strength leads to MTSS and tib post-dysfunctions. Foot exercises during load bearing reduce navicular drop. Barefoot walking and running should be added to training plans.	S F G

Vincent, Brownstein and Vincent 2022 [87]	Narrative review	Trail runners	Injury prevention strategies most applicable to trail runners.	Education needed to interpret pain. Gait retraining using real-time feedback. Ensure flexibility via dynamic warm-ups and include injury prevention such as FIFA 11+ and running drills. Strengthen kinetic chain, foot/ankle, balance and plyometrics. Injury prevention ongoing, not season specific.	E G T S

Warden, Davis, and Fredericson 2014 [88]	Narrative review	Not specified	This paper aimed to discuss the literature on optimal loading in a variety of situations in the prevention of bone stress injuries.	Strategies to preventing bone overload: Monitor RED-S; use plyometric/strength exercise, periodization, rest periods from running; avoid early sports specialization; ensure multidirectional training e.g. ball sports during youth; gradual training loading, progress duration before intensity; increase cadence or softer landing; load monitoring via clinicians, coaches, athletes or wearables.	E S T G R

Willy 2018 [89]	Expert view	Not specified	This masterclass aims to outline the use of wearables for both the management and prevention of RRIs	Wearables (watch, insole, or attachment ankle or shoe) provide real-time feedback such as training loads—acute:chronic workload ratio/foot strikes per run and loading per step, gait retraining such as cadence, cumulative loading cycles. Currently, devices are limited in quantifying biomechanics.	G T

Wu et al. 2014 [15]	Systematic review meta-analysis	Endurance—recreational to elite	The effects of exercise-based injury prevention programmes on RRI reduction in endurance runners.	Exercise-based injury prevention programmes did not have positive effect on injury rate, although better effects were found if the intervention supervised and included foot and ankle strengthening.	S

*Note:* Gait retraining (G), strength training (S), training programmes and load management (T).

Abbreviations: BMI, body mass index; E, education; F, footwear; HCP, healthcare professionals; MTSS, medical tibial stress syndrome; R, recovery; RCTs, randomized controlled trials; RRI, running-related injury.

**Table 3:** Group 3 prospective and retrospective cohort studies, surveys and qualitative studies on injury risk reduction in runners.

Author date	Type of study	Population	Main aims and objectives	Thematic analysis			
				Key outcomes	Codes	Subthemes	Theme
Abran et al. 2022 [90]	Survey via Delphi method	Coaches of endurance runners, recreational to elite runners	Coaches' perceptions on changing foot strike in runners—how and why. Comparisons between different foot strikes.	Coaches change foot strike to prevent injury Coaches feel midfoot strike is best for reducing injury/increasing performance. Coaches recommend changing foot strike alongside foot strengthening and running drills. Coaches feel changing cadence and footwear are less likely to change foot strike	Coaches Changing foot strike Cadence Running drills Foot strengthening	Changing gait to midfoot strike is recommended by coaches for injury prevention	Professional guidance and supervision Runners and coaches' welcome knowledge on strategies to reduce injury, but need supervised guidance on best practice.
Blagrove et al. 2017 [91]	Online survey	Competitive middle- and long-distance runners	To identify the extent to which distance runners engage with S&C and the characteristics of those who participate in various activities. The study also aimed to examine whether reported injury rates relate to the training behaviours of runners.	S&C is performed by runners separate to running, before running or afterwards. S&C incorporates all exercise-based activities, e.g., stretching, resistance training, core stability, body weight exercise, running drills, etc., but stretching is most common activity. Taking part in S&C activities has not been associated with lower injury rates.	S&C When to take part in S&C Injury rates	S&C encompasses any exercise-based activity and stretching is most common.	
Souza Junior et al. 2022 [92]	Cross-sectional questionnaire	Street runners	The questionnaire assessed runners' knowledge of gait retraining for reduction of injury, and their preference for delivery—supervised or partially supervised, for example, (1) 8 sessions over 2 weeks supervised in clinic or (2) partially supervised 2 clinic sessions and 6 home sessions.	Most runners are unaware of gait retraining programmes Runners with previous knowledge are more likely to have had a previous injury. Runners believe supervision is needed for gait re-education, but are happy with partial supervision	Gait retraining Runner's previous knowledge Supervision needed	Runners unaware of gait retraining unless have been injured. Supervision requested by runners	
Linton and Valentin 2020 [93]	Survey	Running coaches	What pre- and post-run training practice or strategies do coaches use and which do they believe are effective for reducing injury risk	Coaches predominantly provide advice on training errors and footwear for injury risk reduction. Coaches nearly always include a warm-up and cooldown, but less likely to incorporate prehabilitation exercises into training. Prehabilitation would be included if coaches were given knowledge of best practice.	Coaches advice Training errors Footwear Warm-up and cooldown. Knowledge Best practice	Similar to the practice of warm-ups as part of training, coaches would include prehabilitation exercises if they had knowledge of best practice	
Sugiara et al. 2022 [94]	Retrospective cohort	Collegiate competitive sprinters	Supervised submaximal, maximal and supramaximal running plus hamstring prevention intervention.	Inducing muscle fatigue through supramaximal runs improves effectiveness of injury prevention programme.	Supervised Training Injury prevention programme	Supervised strength programme/training programme reduced injury	
Taddei et al. 2018 [95]	Feasibility study for RCT	Recreational	Supervised weekly foot core strengthening exercises versus stretching for 8 weeks. Unsupervised thereafter.	Participant satisfaction is similar between groups. Initial adherence 87% and reduced to 83% when unsupervised. More information on purpose and benefits of each exercise would help practice the foot exercise protocol.	Supervision Adherence Information	Supervision of exercises. Education on importance of exercises needed.	

Martins de Oliveira et al. 2022 [96]	Qualitative	Combination Stakeholders: runners, coaches, HCPs, physicians and researchers	Three focus groups/meetings with runners and stakeholders involved in developing the RunIn3 RRI injury prevention programme. Aim was to investigate what facilitators helped develop the programme and what barriers arose.	Developing an injury prevention programme is facilitated by a group's enthusiasm. Barriers were people not attending meetings, a lack of moderation during the meetings and ramblings of participants	Enthusiasm Attendance	Injury prevention programme development requires interest in the subject and someone chairing the meetings.	Education Runners welcome HCPs and coaches delivering and providing education on injury prevention strategies ideally supervised, or through educational module
Dhillon et al. 2020 [97]	Online survey	Runners and HCPs	Questionnaire given to runners and HCPs on footwear and injury prevention. Following completion of questionnaire, runners and HCPs completed an educational module and their perceptions following this collated.	Runners' value comfort when selecting footwear, followed by injury prevention then performance. They most commonly went to HCPs for advice HCPs disagreed over the relationship for injury prevention and cushioned shoes, minimalist shoes and heel to drop height Following an educational module 62.3% runners and 54.8% HCPs changed their perceptions	Footwear Disagreement on injury prevention Educational module	Educational module effective for changing perceptions on footwear.	
Fokkema et al. 2019 [98]	Cross-sectional survey	Recreational	“Implementation” questionnaire distributed to participants of INSPIRE trial 7 months “after” running event. Themes on factors important for RRI prevention/usefulness of injury prevention information/whether searched for injury prevention info/barriers and facilitators. Runners not performing RRI prevention practices were asked how they preferred to receive information on RRI prevention.	Women preferred to get information on injury prevention from a trainer or running store. Men prefer to get information from a website or email Runners with previous RRI found injury prevention strategies were more useful than uninjured runners. Most accessed strategies are training schedules, warming up and cooldown.	Information Men prefer online information Women prefer face to face Most likely to want information if previously injured	Giving people options where they obtain information from is important for injury prevention. Runners with previous injury most likely to use injury prevention strategies.	
Johnston t al. 2020 [99]	Qualitative	Recreational	20 women with and 20 women without stress fractures were interviewed via semistructured interviews. Those who had BSI were asked about their perspective on factors related to their BSI, their experience with medical practitioners and changes post-BSI. Those without BSI were asked about factors related to bone health.	Women with BSI increased training more quickly, had poorer nutrition, did not do as much cross-training and continued to run despite warning signs of injury, compared to women without BSI. Belonging to a group was positive for social/mental well-being/sense of belonging, but negative for pushing too hard. Recognizing individual needs is important—stay within own ability.	BSI Lack of nutrition, cross-training Ignoring injury Group helpful Ability	Future injury prevention programmes should involve an education plan on respond to pain. Belonging to a group was positive, but stay within own ability	
Kemler, Valkenberg and Gouttebarge 2022 [11]	RCT	Novice	To investigate personalized online Runfitcheck programme versus runners continuing usual training	After 5 months, the intervention group was more likely to search for information. Runfitcheck made a 10% favourable difference in injury prevention behaviour	Injury prevention online searching	Personalized online programme can influence injury prevention behaviour.	
Linton, Barr and Valentin 2022 [100]	Qualitative	Recreational	Runners completing prehabilitation workshops took part in focus groups. Workshops had multifactorial approach—education loading, recovery, warm-ups, cooldowns, gait retraining, neuromuscular/strength training exercises. They investigated runner's motivations to participate, what gained and barriers to participation prehab.	Runners are motivated to seek information on prehabilitation due to previous injury, and the need to continue running. Runners welcome all information, educational and exercise-based. A workshop approach provides supervision, a group environment and chance to speak to someone face to face.	Motivation to seek information Education welcomed Group environment Supervision	Holistic approach to injury prevention welcomed by runners. A face-to-face workshop provides supervision and social environment.	
Mann et al. 2024 [101]	Survey	Competitive adolescent youth runners on a youth talent programme and coaches	Both surveys were given to runners and coaches and had the same subjects: (1) knowledge; (2) current behaviour; (3) their need and support for RRI prevention measures; and (4) their views on possible content and form of RRI prevention measures.	Reducing injury important for coaches and runners. Coaches accounted growth and maturation, and too little running and poor strength as factors related to injury. Runners thought injury related to overtraining, too little recovery and growth and maturity. Coaches prefer to supervise their runners with injury prevention strategies, whereas runners preferred to do this at home. Runners preferred information from coaches.	Reducing injury key for both runners and coaches Supervision Information	Coaches are key to providing injury prevention strategies to runners.	
Salzler et al. 2016 [102]	Prospective cohort	At least 20 km per week	In investigate runners use of 5-toed shoes/Vibram minimalist shoes when provided standard information.	No runners complied with industry-recommended guidelines for transition to minimalist shoes 86% runners sustained an injury	Lack of compliance Industry guidelines	Despite guidance, runners did not comply with recommended transitioning to minimalist shoes.	
Verhagen, Warsen and Silveira Bolling 2021 [103]	Qualitative	Recreational	The aim of this study was to find out recreational runners' perspectives on injury management and prevention.	Runners believe a small pain is not an injury. Being part of a running group influences decisions to run through pain as well as performance goals. Management of injury was influenced by experience, caused by overloading and not resting, and managed by training modification. Participation in injury prevention was not a conscious thing other than to change shoes regularly, use a training schedule or do core exercises. Runners look to trainers and physiotherapists for expert advice on the prevention of injury.	What is an injury Running group decisions Experience Expert advice from HCPs and coaches	Runners participating in injury prevention strategies do so unconsciously, awareness shoes and training schedules are important. Few take part in active injury prevention strategies. Runners look to HCPs and trainers for injury information.	

Chowdary et al. 2024 [104]	Cross-sectional survey	Recreational and elite long-distance runners	This study aimed to investigate the use of running-related technologies/fitness technologies (Garmin etc) and their relationship with running-related injuries on elite and recreational long-distance runners.	Runners use technology to inform training decisions. Those accessing running technology are more likely to be injured, but also more likely to be an elite runner. Authors suggest these technologies should also incorporate biomechanical and psychosocial factors to enhance injury prevention	Technology Injury Personalization	Runners using technology are *more* likely to be injured. Technologies need to be more multicentred	Technology Technology welcomed by runners, but need be more personalized to be adopted as an injury prevention strategy
Lacey et al. 2023 [105]	Qualitative	Recreational	This study aimed to investigate runners' views on participating in RRI prevention using running technologies and explore facilitators and barriers to using a wearable sensor and app to monitor running habits and injury.	Runners with a clear interest in injury prevention and previous use of technologies may have a bias towards taking part in injury prevention research. Technologies need to be user-friendly, specific to runners' preferences and flexible.	Technology User friendly Runners' preferences	Runners are interested in running technologies for injury prevention.	

Cohler and Casey 2015 [106]	Retrospective survey	Recreational and competitive amateur runners	Questionnaire asked participants who had changed to minimalist shoes why they had changed their trainers and was it because of injury, did runners sustain an injury after changing or did it reduce their injury, what preparation they had done to transition to minimalist trainers.	Runners change trainers to reduce pain or injury. Similar injury rates are seen for runners adopting minimalist trainers as for runners who reported their pain had reduced when adopting minimalist trainers. Some runners avoiding wearing minimalist trainers through *fear* of sustaining an injury.	Footwear Minimalist shoe to reduce injury Fear	Runners have an individualized response when changing to minimalist trainers.	Beliefs, behaviours and self-efficacy Consider runners beliefs and behaviours when developing injury prevention practices rather than just the strategies themselves
Hofstede et al. 2020 [107]	Prospective cohort. Questionnaire	Marathon	Questionnaire on demographics and injury prevention strategies during unsupervised training.	Those with a ‘substantial' injury had higher rates previous injury, sought more shoe advice and more likely to wear insoles support bandages and K-tape. 51.6% runners had had RRI in the previous 12 months. 17.7% runners had RRI during study	Passive strategies for injury prevention	Runners more likely to address injury with shoes, insoles, bandages and supports.	
Hespanhol et al. 2021 [10]	Prospective cohort. Questionnaire	Trail runners	Questionnaire on injury prevention behaviour to investigate injury in runners accessing online injury prevention advice tailored, with fortnightly contact.	Preventative behaviours were as follows: trail shoes, performing strength and conditioning and core training. Association between intention and preventative behaviour 16 RRI per 1000 h of running	Behaviour Footwear Strength and conditioning	Runners believe shoes, strengthening to prevent injury.	
Hulme et al. 2017 [108]	Modified Delphi technique. Multiple surveys with experts.	Recreational	The development of a model to predict RRI. They used a modified Delphi approach with a series of online surveys given to experts in running injury such as academics (some of whom were runners), health practitioners, athletic trainers and coaches. The aim was to agree on understanding and developing a complex system approach on the prevention of running-related injury.	Feedback from runners on injury prevention should inform decision-making. Runners and stakeholders should be responsible for the development of injury prevention strategies. Wearables and digital platforms provide feedback. RRI development is multifactorial, and runners belief and behaviours influence this. Efficacy educational programmes and behavioural change are based on the availability of resources.	Feedback from runners, wearables Multifactorial Availability resources	Involve all stakeholders in injury prevention strategy development. Consider runners' beliefs and behaviours	
Neilsen et al. 2014 [109]	Prospective cohort	Novice	To investigate unsupervised self-devised running programme of runners progressing weekly distance < 10%, between 10% and 30% > 30%.	Runners increasing running distance by more than 30% over a 2-week period were more at risk of injury than if they increased by less than 10% over the same period No statistical difference in injury rates between groups.	Distance Self-devised programmes	Less likely to be injured if weekly distance increases by less than 10%.	
Nguyen et al. 2024 [110]	Feasibility study pilot RCT	Novice	To investigate if volume running replaced by strengthening or recovery reduces injury. 2 supervised weekly sessions and one unsupervised session.	Neither replacing running with strengthening or recovery reduced injury in either group.	Running volume Conditioning	Running should not be ‘replaced' with injury prevention strategies, but additional to.	
Peterson et al. 2022 [111]	Qualitative	Recreational	To investigate male runners' perceptions and views on the influential factors leading to RRI, and prevention and management of injury	Themes developed were self-management for reducing RRI, conditioning needed for reducing RRI and education needed from coaches and HCPs on training loads. HCPs should respect the runner's autonomy, and aim to support their decision-making by providing educated knowledge. Online resources should be provided by HCPs	Self-management Education coaches Runners autonomy Online resources	Resources and education needed from HCPs and coaches on injury prevention strategies, with the consideration of runners' viewpoints	
Wilke, Vogel, and Vogt 2019 [112]	Survey	Recreational	The authors aimed to investigate runners' perspective of MSK pain, and knowledge of injury prevention when preparing for a 3.5-mile race.	Runners preparing for a 3.5-mile race had overall low physical activity levels. A fifth started the race with some musculoskeletal pain. Runners believe stretching and footwear to be most effective strategies for injury prevention. Less than a third of runners thought resistance training and balance to be effective for injury risk reduction. Authors suggest event organizers should provide runners with injury prevention information.	Physical activity levels Beliefs on stretching and footwear Beliefs on strengthening Information needed	Runners have low physical activity levels and many have pain. Runners have beliefs stretching and footwear most effective, and resistance and balance less effective. Organized running events should have injury prevention information.	
Zhao, He, and Liu 2022 [113]	Questionnaire	Recreational	The questionnaire investigated the use of sports protective equipment in runners.	Lack of stretching and excessive training loads was thought to be related to injury. Runners tend to use passive strategies such as supports, and foam rollers to prevent injury	Beliefs stretching, training and passive strategies.	Runners are more likely to adopt passive strategies for injury prevention.	

Bertelsen et al. 2018 [114]	Prospective cohort	Novice obese	To investigate distance, speed, duration and frequency in overweight runners. Unsupervised programme	Obese runners ran slower in first week Obese runners ran shorter distances for first run	Overweight runners	Obese runners at risk of injury if following same novice programme as runners' normal weight.	Injury risk profile.
Buist et al. 2010 [115]	Prospective cohort	Novice and recreational	Runners followed a graduated running programme. Unsupervised runs plus supervised training clinics	Males more prone to RRI are younger and lacked experience. Females prone to injury had higher BMI, lacked running experience, had not previous done an axial sport, e.g., hockey. 25.9% runners sustained an injury.	Experience BMI Lack of axial sport	Males and females have different injury risk profiles. Playing an axial sport can be preventative for injury.	
Damsted et al. 2018 [116]	Prospective cohort	Half marathon	To investigate pace and injury in runners self-selecting one of 3 unsupervised training programmes.	Fewer injuries in more experienced runners, higher pace, than less experienced runners and lower pace. 25.9% runners sustained an injury.	Experience Pace	Experienced runners less injured. Higher pace reduces risk of injury.	
Hamstra-Wright et al. 2013 [117]	Prospective cohort	Marathon	Investigation of unsupervised training programmes for beginners, intermediate and advanced. Questionnaire on injury prevention.	40% runners who incorporated tempo or interval runs in first 6 weeks of training were more likely to sustain an RRI.	Pace	Running too fast early in marathon training increases risk of injury	
Hutson et al. 2021 [118]	Survey	Distance runners	A survey was distributed to female distance runners, asking their training habits (competition level, training and participation in plyometric training), their menstrual function and whether they had had a BSI within the previous year.	Plyometric training was not found to be effective in reducing plyometric training. Runners with menstrual disturbances/oligo/amenorrhoeic runners were more likely to experience BSI and controlling this with hormonal contraceptive could reduce likelihood of BSI.	Plyometric training Menstrual disturbance	BSI not influenced positively by plyometric training. Identify menstrual disturbances and managing this likely to reduce likelihood BSI	
Kluitenberg et al. 2015 [119]	Prospective cohort study	Novice	Investigated via questionnaire factors related to RRI during a graduated running programme, x1 weekly supervised session and other sessions unsupervised.	More likely to be injured if older, higher BMI, previous injury and no previous running experience 10.9% runners sustained an injury	Age BMI Running experience	Age, weight, previous injury and experience related to RRI.	
Malisoux et al. 2015 [120]	Prospective cohort	Recreational	To investigate injury rates in single-shoe users versus multiple-shoe users	Parallel use of more than one pair of shoes reduced risk of injury. Participation in other sports reduced risk of injury 33% runners sustained an injury	Shoes Participation in other sports	Participation of other sports protective for injury, and wearing more than one pair shoes.	
Rothschild 2012 [124]	Survey	Combination recreational and elite	The aim of this study was to investigate the participation of barefoot running or use of minimalist footwear in runners—motivators and barriers.	Runners try minimalist shoes to prevent injury. Young males are most interested in the transition. Few runners transition completely to minimalist. Transitioning to minimalist trainers created muscle soreness of the leg and foot. Barriers transitioning: fear of injury and lack of supervision.	Minimalist shoes Males Transition Fear	Runners transitioning to minimalist are more likely to be young and male, and doing it to prevent injury.	
Warden et al. 2022 [121]	Cross-sectional	Collegiate cross-country runners	Investigation of bone architecture in runners that (1) run plus swimming/cycling or (2) ran plus soccer or basketball during youth.	Enhancement bone microarchitecture, in those that played multidirectional sports during puberty	Multidirectional sport	Youth runners should participate in multidirectional sports to prevent future BSIs.	

Abbreviations: BMI, body mass index; BSI, bone stress injuries; HCP, healthcare professionals; MSK, musculoskeletal; RCTs, randomized controlled trials; RRI, running-related injury; S&C, strength and conditioning.

## Data Availability

All data relevant to the study are included in the manuscript or available as Supporting files.

## Supporting Information

Supporting Information 1 Supporting file 1: Search Strategy.

Supporting Information 2 Supporting file 2: Data extraction for all studies.

Supporting Information 3 Supporting file 3: Quality assessment RCTs.
