# Supplementary material for: Running-Centred Injury Prevention Support: A Scoping Review on Current Injury Risk Reduction Practices for Runners
Source: Transl Sports Med. 2025 Feb 25;2025:3007544. doi: 10.1155/tsm2/3007544 (PMC11986186; doi:10.1155/tsm2/3007544)
Supplement: Supporting Information 1 — Supporting file 1: Search Strategy. [file 3007544.f1.docx]

**Supplementary file 1: Search Strategy**

Prior to the main search and on advice from the University Librarian, a preliminary search was conducted using the database EMBASE to develop keywords and phrases. The final literature search was conducted from inception to July 2023, then updated from July 2023 to April 2024 through databases MEDLINE, CINAHL and SPORTDiscus using the following search terms and highlighted in table 1, and results from the search strategy summarized in Table 2:

Run* AND Prevent* OR “injury prevention*” OR “prevention injur*” OR “prevention of injur*” OR prehabilitation* OR prehab* OR “reduction running-related injur*” OR “reduction running related injur*” OR preconditioning* OR “reduction injur*” OR “injury reduction*” OR “sports injury prevention*” OR “reduce running-related injur*” OR “reduce running related injur*” OR prevent* OR reduc* AND Injur* OR “sports injur*”

| 1. **Running** | 1. **Prevention** | 1. **Injury** |
| --- | --- | --- |
| Run* | Prevent*  OR “injury prevention”  OR “prevention injur*”  OR “prevention of injur*”  OR prehab*  OR “reduction running-related injur*”  OR “injury reduction*”  OR “sports injury prevention*”  OR “reduce running-related injur*”  OR “reduce running related injur*”  OR prevent*  OR reduc* | Injur*  OR “sports injur*” |

Table 1: Database Totals

Example Database search SPORTDiscus:

Search modes and expanders: find all my search terms

Apply related words

Limit results to:

Full text

Publication date 2000 to June 30^th^ 2023 and June 2023 to April 2024

Peer reviewed

Language: English

Country: All

Publication type: Academic journal

Database subset: All

Document type: All

| **Database** | **Date Searched** | **Results, n** |
| --- | --- | --- |
| MEDLINE | June 2023  April 2024 | 1857  156 |
| CINAHL | June 2023  April 2024 | 412  43 |
| SPORTDiscus | June 2023  April 2024 | 1201  96 |
| Total 3470  295 | | |

Table 2: Search strategy results
