# Supplementary material for: Running-Centred Injury Prevention Support: A Scoping Review on Current Injury Risk Reduction Practices for Runners
Source: Transl Sports Med. 2025 Feb 25;2025:3007544. doi: 10.1155/tsm2/3007544 (PMC11986186; doi:10.1155/tsm2/3007544)
Supplement: Supporting Information 2 — Supporting file 2: Data extraction for all studies. [file 3007544.f2.docx]

**Supplementary file 2: Data extraction for all studies**

**Table (a): Data extraction for randomized controlled trials, prospective and retrospective cohort studies and feasibility studies.**

| **Author** | **Date** | **Type of study** | **Population** | **Participants** | | | **Intervention** | **Comparator** | **Method of delivery** | **Targeted injury(s)** | **RRI outcomes**  **(%)** | **RRI rates per 1000 hr exposure** | **Statistical significance** |
| --- | --- | --- | --- | --- | --- | --- | --- | --- | --- | --- | --- | --- | --- |
|  |  |  |  | **Age**  **Year (SD)** | **Female (n)** | **Male (n)** |  |  |  |  |  |  |  |
| **Adriaensens** | 2014 | RCT | Recreational  n=214 | 23.9 (4.3) | 152 | 62 | Website with online injury prevention module aimed at modifying behaviour towards injury prevention | Asked to read magazines unrelated to running or injury for 30 minutes | Unsupervised.  Participants to visit website for minimum 30 minutes. An online questionnaire of questions that created a personal risk profile for the runner provided tailored feedback | All injuries related to running. | TP1 effect for all outcome measures compared with control group. At TP2/ 3 months intervention group, effect only for behaviours relating to warm up and frequency of shoe replacement p<0.01 |  | Yes, at TP1 |
| **Baltich** | 2016 | RCT | Novice and recreational  n=129 | 32.8 | 103 | 26 | Group 1. Resistance strength training. 5 minutes warm-up plus 20 minutes resistance strengthening training using resistance bands for 8 weeks.  Group 2. Functional strength training.  5 minutes warm-up plus 20 minutes functional strengthening training using BOSU ball (lunges, squats, hops, jumps, single leg standing). | 20 minutes stretching warm up | Unsupervised  Instructional videotape as well as one-page summary sheets. | Lower extremity or lower back | overall n = 52 (40%).  Resistance group n = 16 (37%),  IR = 31.6 (95% CI 18.4, 50.5)  Functional group n = 21 (48%),  IR = 32.9 (95% CI 20.8, 49.3).  Control n = 15 (35%), IR = 26.7 (95% CI 15.2, 43.2) | resistance group 31.6 / 1000,  functional group 32.9 / 1000,  control 26.7 / 1000 | No |
| **Bertelsen** | 2018 | RCT | Novice obese runners  n=56 | 39.2 (9.5) | 45 | 11 | Novice runners termed "obese"/ BMI 30-35. Intervention 1 was a walk/run programme x3/week over 4 weeks, starting with 3km total per week. | NOT a control  Novice runners termed "obese"/ BMI 30-35.  Intervention 2 was walk/run programme x3/week over 4 weeks, starting with total 6km/week. | Unsupervised  graduated training programme | All muscle, joint, tendons and/or bone injuries | overuse injury 37.5%. 27.6% in reduced group/ 3km, 48.1% in standard group/ 6km | Cumulative risk difference: 31.2% risk of RRI, and 47.8% for overuse injury, for reduced start to run distance | Injury risk was greater if starting a 6km/week running programme compared with 3km/week |
| **Bertelsen** | 2018 | Prospective cohort study | Novice obese runners  n = 914 | 37.7 (10) | 455 | 459 | 3 groups:  Normal. BMI less than 25 kg/m^2^  Overweight. BMI 25 to 30 Kg/m^2^  Obese. BMI 30 Kg/m^2^ or higher |  | Unsupervised.  GPS running watch monitored distance, duration, speed and frequency of running sessions during first week of running programme. | N/A |  | Overweight and obese runners ran a shorter distance, and slower for their “first” session, and during the first week overall slower but distance and duration the same as normal weight runners. | Overweight and obese runners more at risk of injury if following same programme as normal weight runners when starting to run. |
| **Bredeweg** | 2012 | RCT | Novice  n=432 | 38.1 (10.8) | 283 | 149 | GRONORUN  Preconditioning 4 wks then 9 wk walk/run | No preconditioning but followed the same 9 wk walk/run | Unsupervised  preconditioning programme although participants were instructed verbally on hopping exercise and given video instruction on training log | Lower extremity or lower back | Injury rate Precon group 15.2%, and control group 16.8% | Intervention 31 (24.0 to 38.0)  Control 30 (24.0 to 37.0) | No |
| **Buist** | 2008 | RCT | Novice  n=532 | 39.8 (10.1) | 306 | 226 | Graded 13 week walk/ run training programme, building up to 4 miles, progression based on 10% training rule | Standard 8 week walk/run training programme | Unsupervised  Written and verbal information. | Lower extremity or lower back | 20.8% in graded programme, and 20.3% in control group | Graded training 30 (22-38)  control was 38 (27-49) RRI/1000 h running | no |
| **Buist** | 2010 | Prospective cohort study | Novice and recreational  n = 629 | 43.7 (9.5) | 422 | 207 | 8 week graduated running programme for the groingen 4 mile for novice and recreational runners. Investigating RRI during the graduated running programme |  | The graduated training programme was given to participants and at the end of weeks 3 and7, 5 training clinics were organized by local running clubs (?sounds like they were supervised sessions).There were 2 progammes for each the novice and the more experienced runners | Lower extremity or lower back | 25.9% runners sustained a RRI | 30.1 per 1000 hrs | For both males and females no previous running experience was most important risk factor. males - more prone to RRI if younger and had lack of running experience. Females - higher BMI lack of running experience and previous experience in non-axial sport e.g. cycling (i.e they hadn't done an axial sport previously e.g. football, hockey netball). Biggest drop out after RRI was seen in novice runners |
| **Chan** | 2018 | RCT | Novice  n=320 | 33.9 (9.5) | 162 | 158 | 2 weeks, 8 sessions gait retraining progressing from 15 mins to 30 mins, self-selected speed, using visual biofeedback (vertical ground reaction force) to "run softer" | 2 weeks, 8 sessions progressing from 15 mins to 30 mins, self-selected speed, n gait retraining. | Supervised sessions with individualized feedback given for first 2 weeks and none thereafter. | Any running related musculoskeletal complaint | Injury was 16% and 38% in the gait retraining and control groups, respectively. | The hazard ratio between gait retraining and control groups was 0.38 (95% CI, 0.25-0.59), indicating a 62% lower injury risk in gait-retrained runners compared with controls | yes |
| **Cloostermann** | 2022 | RCT | Recreational  N=4050 | 42.3 (12.1) | 1480 | 2570 | online injury prevention programme with 10 items on specific advice and tools to prevent RRI | Continued their usual race running preparation. | Unsupervised  Access to website and app, and fortnightly newsletter | Lower extremity or lower back | 35.5% in intervention and 35.4% in control group |  | no |
| **Damsted** | 2018 | Prospective cohort study | Half marathon  N= 784 | 37 (29-46) | 507 | 277 | ProjectRun21. Runners recruited could select a programme from a choice of 3 (Distance-based schedule; pace-based schedule; mixed schedule) or follow their own self-selected programme of training for half marathon. |  | Runners self-selected their training schedule on registration.  14 weeks | Lower legs | Although not statistically significant, there were fewer injuries in runners categorized as having high experience (RD= -11.3% (-27.2% to 4.6%)) or high pace (RD=-17.4% (39.0% to 4.5%)), and for runners with both high experience and high pace (RD+-8.1% (-22.3% to 6.1%)) compared with runners having low experience and/or low pace. | 136 (20%) runners sustained a RRI overall. | Less likely to be injured if more experienced and higher pace. |
| **Desai** | 2022 | RCT | Recreational  N=433 | 39 (8.6) | 203 | 230 | Strength training x2/week | Continued training as normal. No contact with anyone | Initial contact otherwise unsupervised.  Runners had familiarization period and dialogue with tester as needed, otherwise continued the programme unsupervised using an exercise programme with pictures, instructions and videos | Lower extremity or lower back | No statistical sig between groups for RRI 27.1% control and 23.0% intervention. |  | no |
| **Dubois** | 2015 | Pilot RCT | Recreational  N=24 | 31.7 (8.2) | 17 | 7 | 16-week training programme towards completion half marathon wearing traditional running shoes (provided with one of 8 types) or minimalist shoes |  | Unsupervised | Running-related injury | There were equal rates of RRI in both groups - 25% |  | no |
| **Fokkema** | 2019 | RCT | Recreational  N=2378 | 41.2 (11.9) | 1126 | 1252 | INSPIRE trial  multifactorial online injury prevention programme | continue normal running training | Unsupervised.  Participants sent a link to website | Lower extremity or lower back | 37.1% overall  intervention group 37.5% (95% CI 34.8 to 40.4) RRI compared with 36.7% (95% CI 34.0 to 39.6) in the control group |  | no |
| **Fuller** | 2017 | RCT | Recreational  N=61 | 27 (7) | 0 | 61 | Group 1 RFS - were given conventional trainers  Group 2 RFS - were given minimalist trainers |  | Unsupervised.  Instructed to gradually increase running in new shoes | Pain in foot, ankle, calf, shin, knee, thigh and lower back | 37% runners had a RRI in conventional group, and 52% runners had an injury in the minimalist group |  | No although increased risk of injury if heavier and wearing minimalist shoes |
| **Halvarsson** | 2019 | RCT | Elite orienteers  n = 62 | 24.1 (3.6) | 30 | 32 | Runners in intervention group were given 4 exercises addressing neuromuscular control (single leg stand, runners pose, one leg heel raise and one leg hop) with 3 progressive difficulty levels to be performed x4/week for 14 weeks | Not clear but sounds like they did not get any input | Unsupervised.  Runners were explained and demonstrated the exercises once then unsupervised thereafter with just access to pictures | Lower leg injuries and specifically ankle sprains | 28 injuries in intervention and 36 injuries in control group/no sig difference overall between groups for substantial injuries and ankle sprains. | No significant difference between control and those completing exercises less than x2/week, but there was a significant difference between those completing exercises more than twice a week with subgroup analysis i.e. reduced injury by 64% | No sig difference overall  Subgroup analysis  The more frequently the exercises were performed the better the effect |
| **Hamstra- Wright** | 2013 | Prospective cohort survey | Marathon runners  N = 115 | 20-70 years | 84 | 31 | RRI recorded during preparation for marathon via survey pre-training (all runners were given a 18 week training programme for beginners, intermediate and advanced runners) and filled in a questionnaire pre-training, 6 and 12 weeks into training, and post training/marathon. Questions included training preparation and mental skills preparation.  Runners were not given a specific intervention, but the survey looked at associations with RRI, and injury preventative factors in marathon preparation. |  | Unsupervised – participants followed a beginner, intermediate or advanced training programme. | N/A | Odds of experiencing injury in first 6 weeks was 3.96 higher for those who did tempo running vs those who didn't. No significant difference in mental skills prep between injured and non-injured runners in first 6 weeks, 12 weeks or post-training | Type of running preparation affected RRI in the first 6 weeks of starting the marathon training programme. Almost all of the runners who incorporated tempo or interval runs in the first 6 weeks of training sustained a RRI. | Mental skills set during training did not have any impact on RRI during marathon training but the authors said they looked at this at the start of training, but did not look at how runners used this during training. |
| **Hofstede** | 2020 | Prospective cohort study | Marathon  n = 161 | 40.7 (11.7) | 71 | 90 | Baseline questionnaire at 16 weeks before marathon had questions on demographics (lifestyle factors, training-related factors and running experience) and also what preventative strategies they used whilst training for a marathon - warm-ups, cool-s and/or stretching exercises to prevent RRIs. Had they got advice on running shoes, did they use insoles, braces, bandages, or k-tape, compression socks, and different shoes for different training surfaces to prevent RRIs. OSTRC questionnaire was completed every 2 weeks to calculate a RRI severity score. |  | Unsupervised. To compare whether there was a difference between runners who did or did not use injury preventative measures before training/ at baseline for the marathon and compare with those who sustained minor or more significant injury during training. | All running-related injuries | At base line 51.6% runners reported a RRI in previous 12 months. 74.6% were classified as substantial RRIs. Throughout the study RRI prevalence was 17.7%. 67.9% with substantial RRIs sought advice on training shoes compared with runners with non-substantial RRIs, and they wore more braces bandages, used K-tape more often. | There was a significant difference in the use of shoe advice, insoles and supports between those in the substantial RRI group (who also had higher rates of previous injury in the previous 12 months) and those in the non-substantial RRI which suggests runners may be using this because a previous RRI has not resolved. | 59.6% used at least 1 additional not previously used preventative measures during the 16-week training for marathon. |
| **Hespanhol Junior** | 2018 | RCT | Trail runners  n=232 | 44.5 (9.5) | 75 | 157 | TRAILS study  All participants received the same injury prevention advice at baseline. Thereafter intervention group given “tailored” advice every 2 weeks specific to being put into 3 subgroups (no RRI/ non-substantial RRI/ and substantial RRI). | Given general advice on RRI prevention one week after baseline/ same initial information as intervention group at baseline. No further advice after this. | Online programme, but participants in intervention group were contacted every 2 weeks and given tailored advice adapted to RRI status | All musculoskeletal injuries, integumentary systems (blisters, nails) and concussion | 27.5% of participants in intervention (n=18) vs 26.9% (n=18.8) in the control | Trails reduced risk by 13% | No effect on behaviour towards injury prevention, but was effective in reducing risk of injury after 6 months.  This study suggests online should be supplemented with supervised exercises for effect to really happen. |
| **Hespanhol** | 2021 | Prospective cohort study | Trail runners  n = 232 | 44.6 (9.5) | 75 | 157 | This paper uses data from the TRAILS study - see Hespanohol 2018. All participants received the same injury prevention advice at baseline. Thereafter the intervention group were given tailored advice every 2 weeks specific to being put into 3 subgroups (no RRI/ non-substantial RRI/ and substantial RRI). The questionnaire administered was a preventative behaviour Questionnaire, with questions about the determinants of preventative behaviour on the Ix prevention categories (warmups, cool downs, using specific trail shoes, strength and core training, neuromuscular facilitation training and flexibility training. |  | 6 months | All musculoskeletal injuries, integumentary systems, and concussion |  | 16 RRI per 1000 hrs of running (95% CI: 14,18) | The most used preventative behaviours were using specific trail shoes, and performing strength and core training. There was a small indirect effect for association between intention and preventative behaviour. Attitude has the strongest relationship with intention and preventative behaviour. Subjective norm was the weakest relationship with intention and preventative behaviour. The Theory of Planned Behaviour should be implemented when designing or using programmes for reduction RRI. |
| **Hollman** | 2019 | RCT | Recreational  n=51 | 48.2 | 37 | 14 | Both intervention and control group received same baseline injury prevention advice, then the intervention group received further advice every 2 weeks until week 11. This advice was customized based on no RRI, non-substantial RRI, substantial RRI. | Injury prevention advice at baseline during weeks 1, 2, and 3 same as intervention and none thereafter. | Unsupervised online information | Lower limb | Compared to the baseline questionnaire the number of correct responses increased in the intervention group and remained unchanged in the control, but this was not significantly significant. Although it was concluded there was a positive change seen towards preventative behaviours and knowledge. |  | No sig diff  91% runners reported there were no barriers to practicing the recommended interventions |
| **Jacobsson** | 2023 | Cluster RCT | Youth track and field  N=142 | 12.8 (2.4) | 92 | 50 | The intervention group was given access to a digital health platform which had topics on training, growth and puberty, recovery, injury prevention, injury, illness, and mental health | The control group received an email to say they would get access to the digital health platform after the 16-week study period. No advice during the study. | Unsupervised but was “supported” i.e. through emails.  At the start of the study, the first email provided a description of the health platform and log-in details. After this emails were sent monthly with suggestions on topics to explore on the platform | Lower limb, upper limb, spine, head and face | There were 25% new injuries in intervention group and 41% new injuries in the control group. |  | Yes  Engaging the parents was instrumental to the result |
| **Kemler** | 2022 | RCT | Novice  N=1411 | 38.1 (10.4) | 1025 | 386 | Intervention group was given access to the online platform | Control continued training as usual. | Participants were given access to Runfitcheck and thereafter no further interaction was made with them apart from the behaviour questionnaires. Runfitcheck provides a personalized training schedule, strengthening exercises appropriate to the individuals current physical condition, and warm-up details that incorporate strengthening exercises. The focus was on the runner’s physical condition and their running goal. | N/A | Runfitcheck made a 10% favourable difference in injury-prevention behaviour |  | After 5 months the intervention group were more likely to search for information |
| **Kluitenberg** | 2015 | Prospective cohort study | Novice  N = 1696 | 43.3(10) | 1332 | 364 | NLstart2run study. Participants started a 6 week running programme to build up to continuous running in 6 weeks. Baseline questionnaire and they recorded injuries for the 6 weeks of the training programme. No long term follow-up. |  | x1 supervised session, x1 unsupervised session and a 3rd optional unsupervised session/ week.  6 weeks | Lower extremity or lower back | 185 (10.9%) runners developed a RRI | 27.5 RRIs per 1000 h of running or 9.9 RRIs per 1000 athletic exposures. | 10.9% runners sustained a RRI. Higher age, higher BMI, previous injury, and no previous running experience were related to RRI. |
| **Letafatkar** | 2019 | RCT | Novice/ less than 2 years running experience  N=49 | 32.7 (9.0) | 0 | 49 | CT (conditioning training). CT was based on programme by Brushig et al 2008  CT+feedback (verbal plus mirror) | Sham/ placebo - truck and upper limb exercises 3 x weekly for 8 weeks. | Supervised  All sessions were supervised. Programme x3/week for 8 weeks. | All musculoskeletal injury | Injuries from pre-test to 12 months post-test  CT - RRI reduced 32%  CT with feedback - RRI reduced 64.6%  control - RRI reduced 15.5% |  | Yes, both CT and CT+feedback group showed significant improvement |
| **Letafatkar** | 2020 | RCT | Novice  N=60 | 32.9 (6.1) | 0 | 60 | Neuromuscular training (NMT).  The NMT was based on the Emanvirdi et al 2019 programme  NMT plus knee valgus control instructions (VCIs) | Sham exercises involved upper limb movements and back extension exercises | Supervised  Sessions were supervised x3/week for 6 weeks. | Lower extremity or lower back | Pre-post testing/follow up 12 months:  NMT - 31.58% reduction  NMT plus VCI - 65.53% reduction  Control - 13.46% increase |  | Yes, both NMT and NMT plus VCI showed significant improvement |
| **Lundstrom** | 2018 | RCT | Marathon  N=34 | 20.7 (1.3) | 19 | 15 | 3 groups, all followed the same marathon plan. They all had 8 weeks, Run-In (RI) of 4-5 running sessions per week, before they started the intervention phase (marathon period MT) for 12 weeks. Intervention group 1 had core exercises x1/week.  Intervention group 2 had plyometric exercises, | Control group continued the same marathon plan with no additional exercises. | The long run and high intensity sessions were supervised during the MT phase, but it is not clear whether the exercise interventions were supervised or unsupervised. | Injury and pain | No differences in days missed training, or readiness to run between groups during RI and MT periods.  The core group missed more days due to injury during MT period. |  | No |
| **Malisoux** | 2016 | RCT | Recreational  N=553 | 38.3 (9.7) | 212 | 341 | heel to toe drop shoes of 10 mm (D10)  heel to toe drop shoes of 6 mm (D6)  heel to toe drop shoes of 0 mm (D0) | unsupervised | unsupervised  Wear shoes for all running activities | Lower extremity or lower back | 136 participants (25%) overall had a RRI. D10 21.6%, D6 27.4%, D0 24.6%. | overall incidence was 10.33 injuries per 1000 hrs running (95% CI, 10.28-10.39). | No sig diff.  Although secondary analyses found lower injury rate in occasional runners using lower drop shoes. |
| **Malisoux** | 2016 | RCT | Recreational  N=372 | 40.5 (10) | 148 | 224 | standard shoes heel-to toe drop 10mm  motion control heel-to toe drop 10mm |  | Unsupervised  Use shoes for all running activities | Lower extremity or lower back | There were 32.4% RRI in standard shoes, and 17.6% injuries in motion control group, and runners with pronated feet may benefit more from motion control shoes | Overall incidence 7.69 /1000 h of running | Yes  Significant for motion control shoes |
| **Malisoux** | 2015 | Prospective cohort | Recreational  N = 264 | 42.35 (9.3) | 69 | 195 | Single shoe users  Multiple shoe users |  | To train on average at least once a week, and report participation in other sports | Lower extremity or lower back | Overall 33% runners experienced at least one RRI over the study period. | Overall incidence was 7.64 RRI/1000 hr of running | The parallel use of more than one pair of running shoes was  a protective factor [hazard ratio (HR) = 0.614; 95% confidence interval (CI) = 0.389â€“0.969  The participation in other sports demonstrated reduced risk of injury in runners - i.e., was protective for RRI. The use of alternating shoes reduced risk of injury in runners. |
| **Malisoux** | 2019 | RCT | Recreational  N=848 | 40.5 (10) | 329 | 519 | Soft cushioned shoes  Hard shoes |  | Unsupervised  Runners were instructed to use the provided shoes for all running activities, and to perform at least x1 run/week | Musculoskeletal injury of the lower limbs | 12.6% in soft shoes, 17.6% in hard shoes | Overall, 5.67 per 1000 hrs running (95% CI, 4.78-6.76) | Lighter runners had higher risk in harder shoes. |
| **Mendez-Rebolledo** | 2021 | RCT | Adolescent youth female track and field  N=22 | 15.15 (2.4) | 22 | 0 | The NM exercise programme was carried out following each training session during preseason. | Track and field runners followed their normal preseason programme. | Supervised | thigh muscle strain (quadriceps and hamstrings), knee bursitis, knee tendinopathy, and other bone injuries (medial tibial stress syndrome or stress fracture |  | 17.89 injuries per 1000 hrs athlete exposure in conventional group.  6.58 injuries per 1000 hrs athlete exposure in NM training group (relative risk = 0.38; 95% confidence interval, 0.18 to o.82; p = 0.044. | yes |
| **Neilsen** | 2014 | Prospective cohort study | Novice runners  N = 873 | 37.2 (10.3) | 432 | 441 | Runners used a self-structured running programme, and were categorized into 3 groups based on weekly running distance progressions less than 10%, between 10 and 30% and 30% or more. RRI monitored over 1 year |  |  | N/A | Runners increasing running distance by more than 30 % over a 2 week period were more at risk of injury than if they increased by less than 10% over the same period which was statistically significant. |  |  |
| **Nguyen** | 2024 | Feasibility study pilot RCT | Novice  N = 74 | 20.8 (2.4) | 38 | 36 | Runners completed a 21 week strengthening/running programme x3/week. 2 were supervised and 1 unsupervised. Running volume was replaced with strength training. | Runners attended x2 supervised session and x1 unsupervised and received running and recovery session only (no strengthening) |  | Lower limb | 38% RRI in control group and 16.6% in intervention group. This was not statistically significant |  | No |
| **Ramskov** | 2018 | RCT | Recreational  N=447 | 39.5 (9.9) | 447 | 274 | 8 weeks pre-conditioning/ both groups completing the same programme.  Group 1(S-I): running programme 16 weeks - intensity progressed x3/week  Group 2 (S-V): running programme 16 weeks - volume progressed |  | Unsupervised  Participants were given access to their training programme online and via an app. | Injury of muscles, tendons, joints or bones | There were 60 injuries during pre-conditioning (7%)  Overall there were 80 injuries during intervention  (S-I n = 36/16.3%, and S-V n=44/19.47%)) |  | No sig diff between groups |
| **Salzler** | 2016 | Prospective cohort study | At least 20km per week  N = 14 | 28.86 (6.26) | 6 | 8 | 5-toed shoes - Vibram minimalist running shoes. |  | Participants were given a standard transition information leaflet which is normally included in the manufacturers box. Shoes were fitted face to face.  Duration 12 weeks. | N/A | Pain - average VAS score was 3.6 at 12 weeks  time to injury - average 5 weeks |  | No runners complied with the industry recommended guidelines for transition to minimalist shoes. |
| **Suda** | 2022 | RCT | middle and long-distance recreational runners  n=118 | 41 (7.35) | 57 | 61 | 8 weeks foot-core muscle training including 12 progressive exercises (volume and difficulty) | 5-minute static stretching protocol. | Supervised weekly x1 and 3 unsupervised for 8 weeks then unsupervised thereafter.  Online access to exercise descriptions and videos to perform the same exercises x3/week unsupervised.  Control unsupervised  online descriptions and images to be performed x3/week unsupervised. | Any musculoskeletal injury or pain | 15.7% in intervention group, 32.7% in control group, 23.7 overall |  | Yes  older age and higher training volume were risk factors for RRIs  N/B this is a secondary analysis of the Taddei et al 2020 RCT study |
| **Sugiura** | 2022 | Retrospective cohort study | Collegiate competitive sprinters  N = 613 | Age 18 - 24 | 0 | 613 | The injury prevention intervention consisted of 2 parts:  1. submaximal, maximal and supramaximal running training to acquire maximal running speed and speed endurance using training techniques to induce repeated acute fatigue leading to an adaptive response.  2. Hamstring injury prevention programme. |  | Coach lead supervision  24 weeks | Hamstring | incidence of hamstring injuries:  submaximal and maximal running period 1 - 103.4, period 2 - 131.3, period 3 183.9. no significant change.  during supramaximal running, hamstring incidence was period 1 - 137.9, period 2 60.6, period 3 6.7, a significant reduction. |  | The authors concluded that by inducing muscle fatigue through supramaximal runs, the smaller number of runs can improve the effectiveness of injury prevention programmes. |
| **Taddei** | 2018 | Feasibility study for a RCT | Recreational long distance runners  N=31 | 42.1 | 13 | 18 | 8 week training programme for foot strengthening exercises  Duration 1 year | 5 minutes stretching and warm-up protocol | x1 weekly "group" session supervised by a physiotherapist, with instruction to perform the same exercises x2/week unsupervised.  Control - Unsupervised. no detail on frequency | N/A | 31% of intervention group did not complete all 24 sessions in first 8 weeks, 28% didn't complete from week 8 to 16.  The average adherence rate was 87% between T0 and T8, while  adherence between T8 and T16 was 83%. There was 3% dropout. |  | Participant satisfaction was similar between groups, although expectations were lower in the muscle strengthening group. When asked what would make them practice the foot exercise protocol more often they answered that more information on the purpose and benefits of each exercise would help. Significant increase strength of abductor hallucis, flexor digitorum brevis. |
| **Taddei** | 2020 | RCT | Recreational  N=118 | 40.9 (7.35) | 57 | 61 | 8 weeks foot-core muscle training (hybrid supervised x1/wk/unsupervised x3/wk) including 12 progressive exercises (volume and difficulty). After 8 weeks unsupervised until end of study at 12 months | 5 minute static stretching protocol x3/week.Online descriptions and images to be performed x3/week unsupervised. | Hybrid – supervised x1/wk 8 weeks and x3/week unsupervised. Unsupervised thereafter  Online access to exercise descriptions and videos to perform | Lower extremity or lower back | Overall injury rates were 28 participants (23.5%; 95% CI,16.1%-31.4%), 20 from the control group (16.9%; 95% CI, 10.2%-23.7%) and 8 from the intervention group (6.7%; 95% CI, 2.2%- 11.3%) |  | Yes.  Significant difference reduction RRI between groups but not until between 4 and 6 months. 2.42 fold lower rate of RRI |
| **Thiesen** | 2014 | RCT | Recreational  N=247 | 41.8 (10.4) | 111 | 136 | 1.given standard running shoes with soft midsole  2.given standard running shoes with hard midsole |  | Unsupervised.  Instructions were to train on average x1/week and use the shoes provided for all running sessions but not other sporting activities. | Lower limb, upper limb, spine and head | There were 69 (27.9%) injuries in total. | 12.1 RRI/ 1000 h running | No sig diff |
| **Toresdahl** | 2020 | RCT | First time/ novice marathon runners  n=720 | 35.9 (9.4) | 500 | 220 | Strength training group was given a 10-minute instructional video and a handout detailing exercises which focused on core, hip abductor and quads strengthening with beginner and advanced tracks. | Observation group/ no intervention. | Unsupervised | All injuries | 7.3% (27/368) major injury in observation group and 7.1% (25/352) major injury in strength training group.  50.5% (186) of runners in observation group reported minor injuries, 46.3.% (163) in strength group |  | No sig diff |
| **Van der Does 2023** | 2023 | RCT | Novice  N=741 | 18 - 60 | 496 | 245 | 1.Participants were given access to Runfitcheck on injury prevention information on behaviour change – fortnightly emails.  2. participants were given access to the online Runfitcheck on injury prevention information on behaviour change - once | The control group was not given access to Runfitcheck and advised to continue running as normal. | Unsupervised, but 2 weekly email.  Every 2 weeks this group received a email with a link to Runfitcheck.  participants who completed at least 6 of the 9 health monitors on Runfitcheck were entered into a prize draw. | Any physical complaint. | New running injuries: 23% in fortnightly intervention group (Active Runfitcheck), 26% in single intervention group (Runfitcheck once),  21% in control group |  | No sig diff |
| **Van Hooren** | 2024 | RCT | Recreational  N = 172 | 40.1 (10.8) | 39 | 61 | Runners in intervention group were given pressure sensors which provided real-time feedback, and were provided with instruction on what to do/change.  Duration 6 months | Runners in intervention group were given pressure sensors which provided real-time feedback, and were provided with no instruction on what to do/change | Appears to be unsupervised, but hard to figure out what advice they were given with the wearable (pressure sensor in insole).  Support was provided through the sensor to provide feedback and advice on what to do | Running-related injury | Significant reduction injury in intervention group/10% compared with control/44%. | Per 1000 hrs intervention: 12.6 (6.38-22.4) control: 24.1 (18.1- 32.8) | Yes |
| **Van Iperen** | 2022 | RCT | Long distance runners  N=425 | 44.65 (11.65) | 182 | 243 | Personalized mobile app (REMBO app - Running and Exercise Mental break Optimization app) which measures and provides feedback on psychological factors related to the runners training load capacity. The aim was to provide advice on running -related demands, running resources and recovery in response to the information the runners reported on the app via a traffic light system. | Continued normal running training and did not have access to the app. | Unsupervised.  A personalized approach through interaction with the mobile app. The runners were able to self-regulate their behaviour in response to the app. Details of the app are found in the protocol de Jonge et al 2018.  Only 40.2% used the app at least once. | Injury or bodily damage | Similar injury rates across all time points. Interestingly at T0 which included baseline measurement of injuries within the previous 12 months there were 63% injured in control group and 56.5% injured in intervention which reduced to T5 32.3% control group and 33.6% in the intervention. |  | No |
| **Warden** | 2022 | Cross sectional | Collegiate cross-country runners  N = 32 | 20.8 (1.6) | 32 | 0 | Group 1 - RUN. Cross-country runners +/- swimming/ cycling.  Group 2 - RUN+MDS (multidirectional sports). Cross country runners participating in soccer or basketball before and during pubertal growth period |  |  | N/A | "Female collegiate-level cross-country runners who participated in  MDS (soccer, basketball, or both) before and across their pubertal growth period had enhanced bone microarchitecture (CT scan)  and greater strength at their distal tibia and enhanced bone size  and strength at the diaphysis of their fibula and second MT  compared with runners who solely ran (and did low-impact sports). The strength enhancements ranged from an average  of 11.1% at the fibula diaphysis to 19.5% at the distal tibia.  In those who played MDS, there were also enhancements in  bone microarchitecture at high-risk BSI sites, namely, the base  of the second MT, navicular, and proximal diaphysis of the fifth MT" | the aim of this study was to see whether playing ball sports/ multidirectional sport when developing/ going through pubescence enhances bone architecture in runners | This study found enhanced bone architecture and strength in runners who participated in soccer and basketball before and during puberty, suggesting youth runners should participate in multidirectional sports alongside their running as this could provide them with a more robust skeleton and help to reduce BSI's in the future. |
| **Willems** | 2021 | RCT | Recreational  N=372 | 40.4 (10.5) | 148 | 224 | 1.standard neutral shoes  2.motion control shoes |  | Runners had to run at least x1/week and instructed to wear the trainers during all running activities | Lower extremity or lower back | Runners wearing the motion-control shoes had a lower risk of pronation-related running injuries compared with runners who wore standard neutral shoes (hazard ratio = 0.41; 95% confidence interval: 0.17, 0.98). There was no effect of shoe type (hazard ratio = 0.68; 95% confidence interval: 0.41, 1.10) on the risk of other running-related injuries. | This was data analysed from the original study: Malisoux L, Chambon N, Delattre N, Gueguen, Urhausen A, Theisen D. Injury risk in runners using standard or motion control shoes | Had effect for “pronation related injuries”.  No effect on other RRI’s |

**Table (b): Data extraction for expert opinion papers, reviews, systematic reviews and meta-analysis studies.**

| **Author** | **Title** | **Type of study** | **Population** | **Main aims and objectives** | **Themes, synthesized themes, frameworks** |
| --- | --- | --- | --- | --- | --- |
| **Andreyo** | 2022 | Review | Not specified | The review aimed to review the literature on minimalist footwear and provide recommendations on who is suitable for this footwear, and how to transition to minimalist footwear without injury | Progressive training and an adaptation period is needed. Runners with higher BMI are at increased risk of injury. Female runners especially those with previous stress fractures are higher risk for injury. Runners with previous injury are higher risk. Runners with history of hip, knee and shin injuries may have reduced risk in minimalist footwear, but need to be conscious a change of loading can increase risk elsewhere (Achilles and metatarsals). Clinicians should help runners make an informed decision when changing to minimalist footwear. As well as a graduated transition, gait re-training (run softly, cadence, and strengthening exercises need to be considered. |
| **Alexander** | 2022 | Systematic review with meta-analysis | Novice and recreational | Aim of the systematic review was synthesise and critically appraise the evidence for injury prevention and management of knee injuries in runners and evaluate their effectiveness. | Low certainty evidence (1 trial) for reduced risk of knee injury for running technique re-training. Very low certainty to low certainty evidence (17 trials) for footwear. Online injury prevention programmes have little effect on knee injury risk, and multicomponent exercise, graduated running programme, prevention education programmes do not reduce knee injury risk. |
| **Barton** | 2016 | Mixed methods: Expert opinion/ qualitative, systematic review | Not specified. ?all runners | A systematic review of the literature on studies with gait retraining interventions, and their effect on biomechanical changes and management of lower limb injury, and injury prevention. To explore expert opinion via semi-structured interviews. | Although the study focuses on injury management, the experts interviewed in this study thought gait re-training played a role in injury prevention despite lack of evidence in the literature. They thought reducing vertical loading rate a factor in reducing RRI. Higher cadence and FFS/MFS was considered better for runners. |
| **Blagrove** | 2020 | Expert view/ opinion paper | Adolescent endurance runners | Briefly review the literature that has investigated the efficacy of strength training (ST) on the determinants of endurance running in adolescent runners, and  provide guidelines for best practice to improve performance and minimize the occurrence of overuse injury. | For the youth endurance runner, adding ST sessions twice  per week that includes RT, PT, and sprinting is likely to provide benefits to RE and maximal sprint speed that translate to improved performance.  Moreover, these activities, plus movement skills training (MST) and specific strengthening of tissues vulnerable to injury, are important for lowering the risk of overuse injury. A micro cycle is recommended for training, and it is suggested S&C is performed separately or in the final part of a running session. |
| **Brumitt** | 2009 | Opinion paper/ expert view | Cross country runners (high school endurance athletes) | A review on risk factors for RRI and strategies to address these include a strengthening programme for core and hip muscles to reduce risk of injury in female cross-country athletes. | Female cross-country athletes have a greater risk of injury than do their male counterparts.  There is growing evidence suggesting a relationship between dysfunctional hip muscles and running injuries.  Injury prevention programs for the female cross-country athlete, including core and hip exercises, may reduce the risk of injury, and the authors presents a table of suggested strengthening exercises with frequency and repetitions. |
| **Byrne** | 2014 | Editorial/ expert opinion | Cross country, track and field | An interview with a coach for University students gaining his perspective on injury prevention from his previous and current experience. | To minimize injury whilst maximizing performance focus on importance of recovery after sessions, alternative exercise and balancing lifestyle (sleep, study, prehab, nutrition);  Individualize training volume - not all runners can cope with the same mileage;  Footwear - shoes are to provide protection. Barefoot running should only be used within training as a foot strengthening exercise;  Running form - correcting some form issues can improve running efficiency and thus reduce injury:  S&C - needs to be part of training, but coaches need to be aware of overload as students are already increasing running volume and have increased academic demands;  Advice to running medical community is to be honest with runners and their coaches about uncertainty. |
| **Corrarino** | 2012 | Narrative review | Recreational | 7% US adults participate in running regularly, but can be at risk of stress fractures. The aim of the review is to discuss pathology, stress fracture risk fractures and the author suggests strategies for prevention. | Prevention themes include training surfaces (softer better), shoes (change after 300-500 miles) and consider shock absorbing orthoses, decreasing speed, strengthening programmes. Strengthening programmes should include periodisation, alternating upper and lower limb body weight bearing activities, gradual build-up of miles. The effect of muscle fatigue/importance of strengthening, 4 -week training session (3 week progressive/1-week high intensity) Optimizing nutrition for recovery especially carbohydrate. Graduated training post-BSI. |
| **Craig** | 2008 | Commentary | Recreational | A commentary based on the systematic review conducted by Thacker et al 2002: Which injury prevention methods are most effective in reducing medial tibial stress syndrome in physically active people | Highlights flaws in the methodology in Thacker et al, and also how old the studies included were. They highlight flaws in intervention studies on injury prevention, but also that many studies have been conducted on the military, not a sports specific population therefore can only be generalized and large number of sporting population needs to be looked at in future studies.  They suggested the literature shows promise for shock absorbent insoles, age of running shoes, control of pronation, graduated running programmes, improve soleus strength. They suggest athletic trainers should still be conscious of these factors in injury prevention despite the lack of literature. |
| **Davis** | 2016 | Narrative review | Not stated | The article is based on the principle that every runner as a threshold for injury, and what this can be for a runner depends on many risk factors. They focus on mechanical structure, and load tolerance, and how these should work together for injury prevention using gait re-training. | They recommend that strengthening alone is not enough without using neuromuscular facilitation to aid transition to function. They discuss using real-time feedback during gait retraining to reduce impact loading/ land softly; transition from RFS to FFS; increased cadence. The authors suggest clinicians should aim to use a natural environment for runners rather than lab/treadmill to retrain gait. Useful feedback techniques are mirrors, accelerometers, and instrumented insoles. |
| **Davis** | 2017 | Review | Not stated | The authors look at the current high rate of RRI and review the literature to establish whether this has been associated with changes in foot strike and footwear. They discuss barefoot running, rearfoot v forefoot strike, high vertical load, footwear. | There is emphasis on gradual transitioning when changing to barefoot running, or FFS to reduce injury. They also recommend strengthening is needed alongside this to address calf and foot muscles. |
| **Doyle** | 2022 | Systematic review with meta-analysis |  | This systematic review and meta-analysis looks at RCTs that have used gait re-training and its effect on kinematics, kinetics, pain, performance and injury | Although they did not find gait re-training to be effective for pain and injury, they report that their preliminary findings indicate that it may be effective for injury prevention, in particular reducing landing impact in healthy runners. However, they report there is no sufficient evidence currently to support this. |
| **Hamill** | 2017 | Review | Not stated | A review of the literature on changing foot strike to forefoot strike or mid foot strike and its effect on running economy; reduction impact peak and loading of vertical component GRF, and reduction in risk of RRI. Section 5 reports on RRI and they could not find any large prospective studies, and other large epidemiological studies were done on military recruits. | The authors conclude that a change from RFS to MFS/FFS and association with reduction RRI is speculative and not supported by prospective studies. They in fact say, that while it may benefit some runners, it is likely to be least beneficial in recreational runners. |
| **Hart** | 2013 | Expert view | Not stated | To describe the principles of barefoot running for reduction RRI. | Suggestions for reduction RRI using barefoot activities. No specific evidence-based management rather it is more a commentary on the recommendation to use barefoot activities (e.g. walking, bare foot gardening) x1 hour a a day to build up strength in foot with the principle this increased strength with reduce RRI. They provide a graduated programme for this as well as advice to reduce shoe support gradually.  No equipment needed. However, if the barefoot practice isn't continued the strengthening effects diminish. |
| **Kemler** | 2019 | Review plus expert meetings and survey | Novice | 3 stages in developing an intervention designed to promote behaviour change for reduction RRI (also included ski injuries, but separated both in the main text).  1st was to identify the problem and economic burden.  2nd was athletes, sports experts and behaviour experts’ views plus literature review, survey, focus groups to identify the interventions, and whom and how should apply these.  3rd was development of Runfitcheck designed to be a visually attractive and simple to use web-based tool, influenced by runners, and pilot tested. | In the development of delivery, it was established from surveys and focus groups that what was wanted for injury prevention was that the information should be easily accessible and simple; that novice runners would be the main group targeted; that the training schedule represented the physical condition of the runner and their running goals, that is provided strength exercises and an active warm-up; it should also be accessible by smartphone/ tablet/ PC.  Next stage will be trialled/ pilot tested with runners to establish injury preventative behaviour, then a larger scaled intervention. The main themes developed were knowledge (advice on injury prevention interventions and strength training was provided through personal tailored exercises sent weekly by email), awareness (risk of RRI) and self-efficacy (there were clear instructions and videos, and exercises were based on the individual runner’s current physical fitness). |
| Krabek | 2013 | Opinion paper/ review | Ultramarathon | To review the literature for preventative strategies in ultramarathon runners for managing injury and illness. Combination of literature and expert opinion. | For prevention RRI there are recommendations in the literature, but no evidence.  MSK/RRI: The authors suggest limiting weekly mileage to <64 km/wk; limiting competitions; gradual increase mileage during training 10-20%; train on same surface as will be competing on; strengthen hip and knee muscles; have appropriate comfortable gear and test before race.  Medical interventions for Exercise-associated collapse (EAC), heat-related illness, EAH.  Foot care/ prevention of blisters: sock layers, manage them as soon as they occur to prevent worsening, taping. |
| **Krabek** | 2019 | Review | Youth distance runners | A review of the literature on various factors related to injury in youth distance runners, with recommendations for injury prevention summarized at the end of the article: growth mediated factors, strength, cardiopulmonary, cognitive/behavioural/emotional development, bone health, female athlete triad/RED-S, burnout, training, previous injury, sports specificity, footwear and technique. | The authors deliver recommendations for injury prevention based on their rev of the literature and devise a "readiness of running list" to reduce risk of injury.  1. base training programmes on maturity rather than chronological age.  2. screen for previous injuries and address/ ensure all have strength and neuromuscular training programmes/ multidirectional and impact activities.  3. avoid early sports specialization.  4. x1 rest day/week.  5. flexible light trainers.  6. train on multi surfaces.  7. ensure adequate calorie intake.  8. only compete if following acceptable training programme and as long as healthy weight and calorie intake |
| **Jaen-Carillo** | 2021 | Narrative review | All runners | musculotendinous stiffness in the lower limb is used for optimizing performance reducing injury in runners and this review sets out to discuss how. | Coaches should adapt specific strength training from basic -simple to specific-complex exercises to reduce risk of injury neuromuscular training incorporating eccentric and isometric increases muscle stiffness. Plyometric training is important for stretch shortening cycle specifically the calf complex. Foot strength training helps stiffen the foot and arch during propulsion in running. |
| **Johnson** | 2003 | Opinion paper | Novice and recreational | This 2003 study reviews the literature, but they state the literature at this time in injury prevention comes from expert opinion. However, they emphasise the importance of addressing risk factors for different RRI's to prevent occurrence: foot types and orthotics; running shoes for different foot types; leg length discrepancies and heel lifts; strength and flexibility; training surface. | There is emphasis on the complexity of RRI. What is the risk factor for 1 runner is not the same for another runner. They state addressing intrinsic and extrinsic risk factors is the most obvious way of reducing risk, but every runner will have a different risk profile. They have some ideas currently outdated like shoes for specific foot types. A more novel way to reduce injury risk in runners which they introduce and most likely to be more effective is educating the runners to recognize signs of injury earlier. |
| **Mai** | 2023 | Scoping review | Not specified | A scoping review to look at the design features of running shoes and how individualization of footwear can reduce RRI based on biomechanical risk factors | They found that footwear design features (FDF) may be related to increasing the risk for one injury related to a biomechanical risk factor, but decrease risk of injury for another biomechanical risk factor for another injury. Thus, suggesting that footwear needs to be individualised and specific to injury risk profiles. They reported that certain FDF bear the potential for individualisation and for reducing injury-specific BRFs, but it is not recommended to individualise multiple FDF at once. |
| **Malisoux** | 2020 | Narrative review | Recreational | The aim of this literature review is to provide clinicians and coaches with evidence-based information on choice of shoes to reduce RRI that they can advise their runners with. | A review of the literature on different characteristics of running shoes and their influence on RRI risk.  The research shows that certain subgroups of runners can benefit from different types of shoes. Suggestion for injury preventative strategies based on the literature  1. most standard shoes provide a minimum motion control and this is relevant for reduction injury especially in those with pronated feet  2. cushioning has an injury prevention effect especially in lighter runners  3. comfort is important  4. alternate shoes to avoid overload  6. transition slowly to new shoes  7. runners are individuals and adapt to any given shoe type  8. overall the role of shoe in injury prevention is more complex than the shoe alone |
| **Murphy** | 2013 | Review | Endurance | Review of the literature on the subjects of kinematics; minimalist shoes versus cushioned shoes; RFS, loading and leg stiffness; orthotics; barefoot running shod or unshod. | concludes that switch from shod to barefoot running must be graduated; gait patterns by shod and barefoot runners are significantly different but there isn’t evidence to show if this can reduce injury rates; kinematics also differs between shod and barefoot but with no association with injury. Conclusion is that there is no prediction how a runner will respond to changing from shod to barefoot as injuries are multifactorial, but the advice is from this study that if the runner is not presently injured there is no evidence that changing to barefoot running will prevent them getting an injury. |
| **Napier** | 2021 | Opinion paper | No specified | A causal framework is presented  which encourages injury prevention interventions to not just consider addressing 1 specific factor e.g. such as biomechanics, without considering that an injury won't occur without a training load error. | Key points of advice: training programmes have often focused on training volume, rather than training load, and that injury may often occur when volume remains static but there has been increased loading e.g. hill reps; tailor the programme to an individual’s risk profile (physical, training, psychological); consider whether masters or adolescent; tailor to previous injury e.g. Achilles, PFP; consider all aspects of RED-S and not to neglect energy intake/ routinely screen; use wearable technologies to monitor training loads . |
| **Nielson** | 2020 | Educational review | Combination | A rev of RCTs on running injury prevention research, and the compliance with different interventions. This study reviewed the data from 7 RCTs and 1 experimental study on injury prevention, and looked at compliance, to consider how compliance is reported in injury prevention trials and to consider intention to treat analysis. Compliance was defined as having completed 90% of the intervention given or if referring to running shoes, having worn them for all running sessions. | There were 4 running programme interventions: at the end of the follow up period RUNCLEVER had 0% compliance (Ramskov et al 2016), PR21 0% (Damsted et al 2021), Start to Run 21% (Bertelsen et al 2028), GRONORUN 1% (Buist et al 2007). There were 4 running shoe interventions: Theisen et al (2014) 68%, Malisoux et al (2016) 76%, Malisoux et al (206) 90%, and Malisoux et al (2017) 86%.  Definition of compliance differed across the running programme studies, more experienced recreational runners may be less inclined to chance their running practice than novice runners, and overall,l it is likely runners find it easier to be asked to wear a specific shoe than to be asked to change or adopt a specific running programme, although in the PR2 study runners did have the choice to choose their running programme.  In running trials compliance ranged from 0%-21% whereas running shoes trials had 68%-90% compliance.  Randomised controlled trials (RCTs) in sports injury research: “authors-please report the compliance with the intervention”. |
| **Nigg** | 2019 | Editorial and commentaries | All | This is a series of 6 peer commentaries discussing the main article (Muscle tuning and preferred movement path - a paradigm shift. Nigg et al 2017) which discusses 2 paradigms "impact force" and "pronation control" which they propose should not be used in the context of injury prevention for runners and instead replaced with the runners "preferred movement path". That is, kinematics only change minimally to a change in show and/or orthotics and that it is the runner in the shoe that provides the change. | 1. Becker 2018. New Hypotheses and Unanswered Questions in  Running Injury Research  This author agrees with Nigg et al 2017 and suggests further research should reconsider long held beliefs on impact forces, pronation and RRI.  2. Clark et al 2018 Running impact forces: from half a leg to holistic  understanding  These authors force on the literature on impact force and overuse injury, and acknowledge there limited evidence in quantifying impact force with injury.  3. Federolf et al 2018 A discussion of the Muscle Tuning and the Preferred  Movement Path concepts “ comment on Nigg et al.  These authors conclude more research is needed on orthotic interventions to see if they change kinematics  4. Hamill et al 2018 A paradigm shift is necessary to relate running injury  risk and footwear design  Previous literature has small samples therefore large scale studies are needed to look at design of footwear and injury e.g. Nielsen et al 2014.  5 Paquette and miller 2018 Reconciling new with old injury paradigms and the  need to dig deeper  These authors agree that in fact force and ankle pronation do not provide evidence for injury preventative measures in runners, yet other measures have similarly provided little evidence to date. They suggests tissue loading and adaptation responds to any given stimulus (e.g. footwear, gait changes, training errors) and all should continue to be explored in RRI prevention research.  6. Vanwanseele et al 2018 Muscle tuning and preferred movement path: do we  need a paradigm shift or should we redefine the old?  They agree with Higg et al 2017 that there is little evidence to support these paradigms and future research needed. |
| **Ramsey** | 2016 | Opinion paper | Track/ cross country runners | This paper is aimed to assist coaches, trainers and athletes in track and cross country running to prevent and manage ITB injuries. | Novice runners are more likely to have an ITB problem therefore early injury prevention intervention is needed particularly for this group. Their suggestions are:  Coaches should determine peak mileage for the individual athlete. Running on alternate days can help.  On the principle that faster paced running may aggravate the ITB less, the authors suggest programs should incorporate speed work such as interval workouts, fartleks, tempo runs and gait runs focusing on mid to forefoot strike.  They suggest monitoring the age of the training shoe.  Avoid downhill running  They suggest stretches hip flexors, ITB, and hams, and foam roller |
| **Relph** | 2022 | Cochrane review | All types adult runners | To assess the literature on how different characteristics of running shoes can prevent injury | 12 papers were included in the review. Overall evidence was low to very low certainty, there was variation in definition of running shoe characteristics and too few studies for comparisons.  Conclusions:  - no difference with neutral/ cushioned and minimalist shoes to RRI in recreational runners  - uncertain if motion control shoes v neutral cushioned reduce RRI  - soft v hard midsole may reduce RRI  - recommending shoes based on foot posture index may reduce RRI |
| **Rixe** | 2012 | Opinion paper | Track/ cross country runners | The authors have conducted this review to look at the features of barefoot running and minimalist footwear and their influence on prevention of RRI | The authors conclude that evidence shows that a minimalist running style can have an effect on biomechanics/ kinetics and change foot strike, but this doesn't necessarily lead to reduction RRI's seen in shod running, simply change the type of injury seen.  A slow transition into this minimalist is necessary to prevent injury. |
| **Silva** | 2020 | Opinion paper | Not specified | This paper discusses a holistic approach to injury risk reduction with increasing performance. | The key themes in this paper for injury prevention in runners are:  Importance of sleep for recovery and performance.  Having rest days minimum x1/week, but also active recover/cross training days, especially youth and older runners.  Adequate nutrition and hydration  Mental and emotional health/ Increased stress can negatively affect physical performance. Coaches and HCPs should be aware of their athletes mental health.  Dynamic warm-up and cool-downs.  Lack of strength is a modifiable risk factor - practice gluteal exercise, foot strengthening, core, single leg exercises, resistance and plyometric training.  Overall, ensure adequate recovery from previous injury to prevent further injury. |
| **Stenerson** | 2021 | Opinion paper | Recreational | This paper aims to give a multifaceted approach to reduction RRI which involves a combination of education, and balance and hip strengthening exercises. | The authors recommend that a multifaceted approach to injury prevention education is essential due to the multiple risk factors, and that previous Ix prevention programmes have tended to focus on just one risk factor with limited effectiveness:  Education: Most runners report the main barrier to injury risk reduction practices is not knowing what they should do. professional should educate their runners on risk factors in order to gain greater compliance with Ix prevention interventions.  Functional balance - poor balance is related to falls, reduced performance and poor postural control and single leg static and dynamic exercises with progressions should be incorporated into injury prevention programmes.  Hip strength - Weaker hip abductors are associated with injury in runners and progressive exercises are suggested.  Dynamic warm-ups.  These injury prevention programmes can be incorporated concurrently into in-season training in contrast to periodization training plans/ resistance training that are a focus of off-season. Fitness professionals are best positioned to disseminate this knowledge to runners. |
| **Suc** | 2022 | Narrative review | Endurance runners | The aim of this study is to look at resistance training in endurance athletes and review the effect on running economy, running biomechanics and RRI and thus inform coaches and clinicians to provide specific programmes for their runners | The authors found:  Effect on improving running economy and performance - more effect on running economy was found, particularly in competitive runners and in longer distances e.g. marathon and ultramarathon.  Muscle strength is positively related to performance. No change was seen in body composition, but rather, resistance training effects were due to adaptations of the neuromuscular system.  Plyometric training improved running economy through improved lower leg quasi stiffness and reactive strength as the SSC was better utilized.  Isometric plantar strength training was found to improve tendon stiffness more than plyometric training (Lum et al).  Running technique should be taken into account when designing resistance exercises. as hip strength is important.  Ensure there is foot and ankle strengthening within the programme to reduce RRI risk. |
| **Tenforde** | 2015 | Opinion paper | Youth athletes | This paper looks at the literature on bone health comparing participation in ball sports, and participation in running in youth athletes. | The key themes outlined:  - The time periods for achieving peak bone mass are during childhood and adolescence, and that targeted sports participation and jumping activities are likely to prevent future development osteoporosis.  - athletes participating in ball sports have greater bone mineral density than distance runners.  Excluding the influences of RED-S, the authors propose that youth athletes participating in running, low impact sport (swimming) or the military would benefit from incorporating ball sports or similar activities as a prehabilitation strategy to improve bone health. |
| **Tenforde** | 2020 | Expert based opinion paper | Youth running | This paper aims to provide recommendations for reducing risk of injury in youth runners. | The authors address the following themes:  - participation in higher impact, multidirectional sports such as ball sports for at least 2 years during childhood can reduce risk of bone stress injuries.  - youth runners psychological well-being should be assessed to prevent burn-out, and youth runners should not be encouraged to specialize at this age.  - Youth runners should be screened for low energy availability using the RED-S CAT tools. Early recognition can address and prevent future injury.  - During peak height velocity (growth spurts) monitor training loads. Running training and competition should take individual growth and development into consideration rather than chronological age.  - competitive longer distance events half and full marathons should be discouraged in youth runners.  - youth runners should perform foot and ankle strengthening exercises. Ant transitioning from cushioned to minimalist footwear has to be done gradually and alongside strengthening exercises. |
| **Teyhen** | 2015 | Expert view | Combination | Summary of a study using real-time feedback for running mechanics to reduce risk of injury | Using visual feedback (mirror) can be useful to improve form when cued/ supervised by physical therapists. Audio feedback via use of metronome can be used to improve cadence. |
| **Teyhen** | 2014 | Expert view | Combination | Summary of study by Nielsen et al, 2014. Advice for people starting running or increasing mileage without injury, and perspectives on the commonly used 10% rule. | Overall increasing weekly mileage by over 30% will increase risk of injury. Increasing mileage by less than 10% can reduce risk of injury. Adding speed with distance may make runners more susceptible to patellofemoral pain, patellar and gluteal tendinopathy, ITB and medical tibial stress syndrome |
| **Verhagen** | 2012 | Editorial | Novice | The aim of this paper discusses the health benefits of running, versus the impact of injury particularly in novice runners, and how injury prevention strategies are needed to promote engagement. | The author highlights the lack of studies on RRI prevention particularly in the novice runner who may have a specific injury risk profile.  Suggestions for future RCTS highlight that higher sample numbers are needed for shorter follow ups than longer follow ups, but a sport such as running will have logistical issues with longer follow-ups.  Research is needed to assess overloading of all tissues, specific soft tissues, and underloading.  Overweight novice runners may be at higher risk and this needs to be investigated further.  Running mechanics change with fatigue therefore more research is needed to explore kinematics and RRI prevention. |
| **Vincent** | 2018 | Clinical commentary | Not specified | The aim of this paper is to address focused training of the foot intrinsic muscles for stability and injury prevention. | The authors liken the foot intrinsic muscles to trunk core, where they act to provide stability during single leg activation activities in running.  Poor strength can lead to issues such as MTSS and tib post dysfunctions.  Foot doming with progressions during load bearing exercise is suggested to activate adductor hallucis and abductor hallucis to reduce navicular drop. The authors also suggest barefoot walking and running should be added to training plans. |
| **Vincent** | 2022 | Narrative review | Trail runners | injury prevention strategies most applicable to trail runners. | Education is needed on how to interpret pain such as stop if pain increases, lingers over 24 hours, if pre-existing pain less than 3/10 VAS worsens, or runners begins to compensate. Gait re-training using real-time feedback. Ensure flexibility via dynamic warm-up and include movements from successful injury prevention warm ups such as FIFA 11+, and running drills. Strengthen specific kinetic chain and foot/ankle, balance, plyometrics. injury prevention is ongoing and not season specific |
| **Warden** | 2014 | Narrative review | Not specified | This paper aimed to discuss the literature on optimal loading in a variety of situations in the prevention of BSI's | BSI - due to loading of bone in running exceeding the current ability of the tissues. Strategies to prevent this overload discussed are:  Monitor athletes for RED-S  use plyometric exercise to help generate bone adaptation after rest periods. and use periodization, with rest periods from running (but not other sports).  Avoid premature sports specialization.  Ensure multidirectional loading/ training such as ball sports are encouraged during youth sport, or trail running.  Ensure rest periods when sport is year-round, or gradual increased loading if seasonal.  Ensure gradual training loading, progressing duration before intensity.  Increasing running cadence or softer landing can reduce GRF's  Load monitoring via clinicians, coaches, athletes or wearables.  Improve muscle strength. |
| **Willy** | 2018 | Expert view | Not specified | This masterclass aims to outline the use of wearables for both the management and prevention of RRI's | Wearables (watch, insole, or attachment ankle or shoe) have the ability to provide real-time feedback on data on running kinetics and biomechanics as well as alterations in loading patterns  The main themes identified on the use of wearables were:  Monitoring training loads - acute:chronic workload ratio/ foot strikes per run and loading per step., thus the ability to identify training errors which lead to injury.  Monitoring running biomechanics - currently devices are limited in quantifying biomechanics unless used in lab settings.  Monitoring return to run programmes - as well as walk:run ratios, they can quantify cumulative loading cycles, and prevent further injury.  Monitoring gait retraining - provides feedback e.g., on step rate |
| **Wu** | 2014 | Systematic review and meta-analysis | endurance runners recreational to elite | This study aimed to investigate the effects of exercise-based injury prevention programmes on RRI reduction in endurance runners. | Overall, exercise-based injury prevention programmes did not have positive effect on injury risk or injury rate, although better effects were found if the intervention was supervised. The authors recommend exercise-based interventions should be supervised, performed a minimum of twice a week, and should include foot and ankle strengthening. |

**Table (c): Data extraction for surveys and qualitative studies**

| **Author** | **Date** | **Type of study** | **Population** | **Main aims and objectives** | **Themes, synthesized themes, frameworks** |
| --- | --- | --- | --- | --- | --- |
| **Abran** | 2022 | Survey via Delphi method | Coaches of endurance runners, recreational to elite runners | Coaches’ perceptions on changing foot strike in runners - how and why. Comparisons between RFS, MFS and FFS. | 83% coaches change foot strike to prevent injury.  RFS is seen as the weakest technique (most likely to be related to injury) and MFS the strongest.  MFS is reported as best for reducing injury and increasing performance.  99% coaches also support a change in foot strike alongside ankle and foot strengthening exercises.  The most likely method of changing foot strike is through running drills, then strengthening and mobility exercises. The least likely methods are through a change of cadence and footwear. |
| **Blagrove** | 2020 | Online survey | Competitive middle- and long-distance runners | To identify the extent to which distance runners engage with S&C and the characteristics of those who participate in various activities. The study also aimed to examine whether reported injury rates relate to the training behaviours of runners. | 53% performed S&C activities to improve performance, and 63.1% to reduce risk RRI. Activities were performed either separate to running, as warm-up, after running, part of S&C.  Types of S&C and when it was performed by the runners:  86.2% stretching (after running); 70.2% core stability (either separate or part of S&C sessions); 62.5% resistance training (either separate or part of S&C sessions); 60.4% body weight exercise (either separate or part of S&C sessions); 54.7% foam rolling (separate or after running); 50.4% running drills (warm-up or part of S&C sessions); 31.6% balance(part of S&C sessions or independent); 27.7% movement skills (warm-up or part of S&C); 27.1% circuit training (independent); 14.8% barefoot exercises (independent or part of S&C).  Participation in S&C does not seem to be associated with lower injury rates but higher running training volumes seem to be related to the number of injuries runners experienced. |
| **Chowdary** | 2024 | Cross-sectional survey | recreational and elite long-distance runners | This study aimed to investigate the use of running-related technologies/ fitness technologies (Garmin etc) and their relationship with running-related injuries on elite and recreational long-distance runners. | They found that runners 88% who used running technology used it to inform training decisions, with elite runners accessing metrics such as cadence more frequently than recreational runners. Those runners using running technology most frequently were more likely to be injured than runners using it less frequently which the authors thought may be due to sharing of data and peer pressure through challenges, activity tracking and leader boards possibly influencing runners’ decision making when training. The author suggests running technology should integrate biomechanical and psychosocial factors to enhance injury prevention practices, and use them to guide training decisions. |
| **Cohler** | 2015 | Retrospective survey | Recreational and competitive amateur runners | Questionnaire asked participants who had changed to minimalist shoes why they had changed their trains and was it because of injury. They asked did runners sustain an injury after changing, or did it reduce their injury. They asked what preparation they had done to transition to minimalist trainers. Those runners who had never tried minimalist shoes were asked if they were interested in doing so, and why they hadn't. | 31% of survey respondents had tried minimalist trainers and the most common motivating factor was to reduce injury or pain. 29% who used the minimalist trainers sustained an injury, and 31% reported their pain had improved with minimalist trainers. 35% didn't do any preparation or graduated training to transition to the trainers. Those that hadn't tried minimalist trainers were influenced due to fear of sustaining an injury. Just as many participants sustained an injury wearing minimalist trainers (29%) as those whose pain improved after trying minimalist trainers (31%). The authors recommend minimalist trainers should be introduced with graduated programme and prehabilitation is needed for lower limb strengthening prior to introduction.  41% of those who have never tried minimally shod (n=389) reported being interested in seeing whether minimally shod reduced their injuries. |
| **De Oliveira** | 2022 | Qualitative | Combination runners, running trainers, physical therapists, sports physician, researchers, and stakeholder | 3 focus groups/meetings were conducted in the form of semi structured face to face interviews. Participants (runners, physical therapist, sports physician, nutritionist, researchers) were those involved in developing the RunIn3 RRI Injury Prevention Programme, and the aim was to investigate what facilitators heled develop the programme and what barriers arose.  The following open questions about facilitators and barriers were asked by the interviewer: What did you think about the form of contact? What did you think about the group meetings? Do you believe it was possible to absorb the suggestions of all participants involved in the development process in the way the meetings were proposed? In your opinion, what are the main obstacles to the development of an efficient long-term prevention program? What did motivate you to help developing this program? What factors did you think have hampered the development of this RRI prevention program? Would you have any suggestions/recommendations to the developers of the program? | Overall, there were more reports of facilitators (55.1%) than barriers (44.9%) although there were only 6 facilitators mentioned and 11 barriers. The group meetings were a facilitator as they promoted discussion and ideas, but people not turning up was seen as a barrier, and also "ramblings" and meetings not being moderated were barriers. The groups enthusiasm for the subject was a facilitator. |
| **deSouza Junior** | 2022 | Cross sectional questionnaire | Street runners | The questionnaire assessed runners’ knowledge of gait retraining for reduction of injury, and their preference for delivery - supervised or partially supervised. They were given 2 examples: (1) 8 sessions over 2 weeks supervised in clinic or (2) partially supervised 2 clinic sessions and 6 home sessions. | Most participants overall had no knowledge of gait re-training programmes. Runners that had had a previous injury were found to have higher knowledge of gait re-training, but there was no difference in terms of interest or preference of fully/partially supervised gait re-training programmes. Overall runners believed supervision was an important part of learning gait re-education. The authors feel this is an important choice to give runners to support behavioural aspects e.g. if they prefer supervision they should be offered it. |
| **Dhillon** | 2020 | Online survey | Runners and HCPs’s |  | Initial questionnaire - runners mainly valued comfort, then injury prevention then performance when selecting footwear. HCP's were the runners most common primary source for advice on footwear. There was disagreement amongst HCPs over the relationship for injury prevention and cushioned shoes, minimalist shoes, heel to toe drop height. 1035 participants then completed the educational module and 62.3% of the runners changed their perceptions following this. 54.8% HCP changed their perceptions and 58.7% HCPs said they would change their future footwear recommendations to runners. |
| **Fokkema** | 2019 | Cross-sectional survey | Recreational | An "implementation" questionnaire was distributed to participants (intervention and control) of the INSPIRE trial 7 months "after" their running event. Question themes were on factors they felt important for RRI prevention/ usefulness of injury prevention information/ whether they searched for injury prevention info and what/ barriers and facilitators for RRI prevention/ and runners who didn't perform RRI prevention practices were asked specifically why/ how runners preferred to receive information on RRI prevention. | When runners were asked how they preferred to get their information women preferred to get their information from a trainer or running store. Men preferred websites or e-mail.  Runners with previous RRI rated injury prevention strategies as more useful than runners who had never sustained an injury (76.8%). 81.8% had already performed injury prevention strategies before - most common were changes to training schedule (64.4%) and warming up/cooling down (57.8%). Barriers for those previously injured were not knowing what to do (59.1%). |
| **Hutson** | 2021 | Survey | Distance runners | A survey was distributed to female distance runners, asking their training habits (competition level, training, and participation in plyometric training), their menstrual function, and whether they had had a BSI within the previous year. | Doing 1-2 sessions of plyometric training was not effective in reducing BSI. Menstrual disturbances/ oligo/amenorrhoeic runners were more likely to experience BSI. Controlling menses with hormonal contraceptive could reduce likelihood of BSI. |
| **Hulme** | 2017 | Modified Delphi technique. Multiple surveys with experts. | Recreational | The development of a model to predict RRI. They used a modified Delphi approach with a series of online surveys given to experts in running injury such as academics (some of whom were runners), health practitioners, athletic trainers and coaches. The aim was to agree on understanding and developing a complex systems approach on the prevention of running-related injury. | The complex systems models was developed based on the themes that arose:  Both runners and other primary stakeholders (e.g. physios, coaches) are responsible for the development and prevention of RRI and that feedback from the runners informs decision making; types of feedback can be the use of wearables and digital health platforms; the development of RRI itself is multifactorial with the most common factor being participation in running itself; runners beliefs and behaviours influenced by systemic determinants; efficacy of educational programmes and behavioural change is based on availability of resources |
| **Johnston** | 2020 | Qualitative | Recreational | 20 women with and 20 women without stress fractures were interviewed via semi-structured interviews. Those who had had BSI were asked about their perspective on factors related to their BSI, their experience with medical practitioners and changes post BSI. Those without BSI were asked about factors related to bone health. | The themes identified were:  - ongoing and recurrent MSK injuries. All runners reported some type of RRI. The authors advice runners should be educated on injury prevention.  - activity patterns and training regimens. The authors discuss psychological aspects are involved with over training and need to be considered when managing injured runners nutrition. Education is needed on how to incorporate good nutrition for running into lifestyle.  Prevention and intervention. This was in relation to cross training but didn't manage due to time and fatigue therefore education on balancing running and cross training/ strengthening is needed.  Pain. Education needed on how to interpret and respond to pain.  Mindset - belonging to a group was positive and negative. Positive for social/ mental well-being/ sense of belonging, but negative for pushing too hard. Recognizing individual needs is important - stay within own ability.  Women with BSI increased training more quickly, had poorer nutrition, didn't do as much cross training and continued to run despite warning signs of injury, compared to women without BSI.  The authors recommend that women need more guidance on how to progress running safely; they should incorporate cross training into their training 2-3x/week; they need to adapt their plan for training if fatigued; and shouldn't misinterpret pain that is an injury. Future RRI prevention studies should consider a comprehensive education plan. |
| **Lacey** | 2023 | Qualitative | Recreational | This study aimed to investigate runners’ views on participating in RRI prevention using running technologies and explore facilitators and barriers to using a wearable sensor and app to monitor running habits and injury. | 9 focus groups were held with 27 participants. Incentives (study outputs, evidence-based information and laboratory testing) were discussed as helpful to participating in this research. The authors report that knowledge can influence behaviours on injury risk reduction practices. Participants wanted personal feedback and comparison with their peers either during or at conclusion of the study. It was also found that runners with a clear interest in prevention of injury and/or use of running technologies would be more likely to volunteer for this study, but this could create a bias. providing evidence-based information and laboratory testing throughout the study and ensure participant have been told of the aims of the study during recruitment. The intervention needs to be user friendly technology, and flexible around runners’ schedules and allows them to train with their preferred training plan. Communication should be flexible and specific to each participants preference. Runners overall appear interested in using running technologies for research purposes. |
| **Linton** | 2020 | Survey | Running coaches | what pre and post run training practice / strategies do coaches and RGLs use and which do they believe are effective for reducing injury risk | The most common injury prevention advice given to runners was on training errors and footwear. Most performed warm-up and cool-downs but prehab exercises were included less as part of training. Most coaches would include prehab, but lacked confidence and knowledge of what is best practice. |
| **Linton** | 2022 | Qualitative | Recreational | Runners that had completed a prehabilitation workshop for runners took part in focus groups.  The workshop consisted of 4 supervised sessions over 4 weeks and involved a multifactorial approach for reduction RRI, with both education on strategies such has load management, recovery strategies, warm-ups, cool-downs, and gait re-training covered as well as neuromuscular and strength training exercises.  They investigated what had motivated runners to participate in the workshop, what they had gained, and what barriers they perceived to ongoing participation in prehab. | Themes identified from the interview:  Motivators - novelty of prehab, constant cycle of injury, running becoming unnatural, having the opportunity to speak to someone, running longevity.  Developing awareness and understanding ix prevention - familiarity with concepts, running cues, specificity of exercises, pain monitoring, NMT.  Empowerment of support delivery - supervision, group size, length of workshop, holistic and structured approach, seeing positive changes, supporting literature, convenience of prehab, personal responsibility.  barrier to prehab - volume of information, equipment, time management, when to do prehab, peer influences, lack of supervision.  Overall, the holistic, supervised approach was endorsed by runners with both exercises and education equally important. |
| **Mann** | 2024 | survey | Competitive adolescent youth runners on a youth talent programme, and coaches | Both surveys were given to runners and coaches and had the same subjects (1) knowledge; (2) current behaviour; (3) their need and support for RRI prevention measures; and (4) their views on possible content and form of RRI prevention measures. | Both coaches and runners felt reducing injury was very important. Differences on where injury arose from differed: coaches thought growth and maturation, and too little running and poor strength, versus the runners thought too much training and too little recovery as well as growth and maturity. Both coaches 96% and runners 100% felt injury prevention was very important, but less said they would adopt them: coaches 72%, runners 77%. Coaches suggest Ix prevention should be done at training, but runners preferred to do it at home. Preferences for receiving injury prevention information was mainly from coaches rather than online and websites. They were happy for it to be delivered by coaches, S&C coaches and physios |
| **Rothschild** | 2012 | Survey | Combination recreational, and elite | The aim of this study was to investigate the participation of barefoot running or use of minimalist footwear in runners - motivators and barriers. | The authors found that overall, 37.8% of runners had sustained a RRI in the previous 6 months.  21.9% had tried barefoot running and 30.4% had tried minimalist shoes. The most common reason for using minimalist shoes was to prevent injury (34.3%). Other reasons were advice from friends (24.5%) and books (24.5%). However, 43.6% of these runners used both regular running shoes and barefoot/minimalist footwear, with only 5.8% having replaced them altogether.  Those most interested in transitioning to barefoot/minimalist were younger male elite runners.  Any adverse reactions related to transitioning to barefoot/ minimalist footwear was muscle soreness of the leg and foot, and most reported no ongoing problems (83/1%).  Both fear of injury (54%) and lack of supervision (23.4%) were reported as barriers to transitioning to barefoot/minimalist footwear, but welcomed supervised instruction by coach or running professional.  The authors recommend supervision when transitioning to barefoot/ minimalist footwear, and evidence-based programmes to support the runner. |
| **Peterson** |  | Qualitative | Recreational | To investigate male runners’ perceptions and views on the influential factors leading to RRI, and prevention and Mx of injury | 3 themes were identified:  Mind - self-understanding and management to avoid RRI. Monitoring RRI.  Body - reducing injury requires strength and fitness. Lack of conditioning is related to RRI  Education - belief on coaching needed to structure training and training loads to prevent RRI. Footwear age was thought to be related to RRI, and sudden changes running environment.  HCP's should respect the runners autonomy, and aim to support their decision making by providing educated knowledge. If online sources are a runner’s preferred way of finding information HCPs should provide online sources to assist them.  The authors suggest that when designing an injury prevention intervention, runners viewpoints should be considered, and this can provide compliance to programmes. |
| **Wilke** | 2019 | Survey | Recreational | The authors aimed to investigate runners’ perspective of MSK pain, and knowledge of injury prevention when preparing for a 3.5-mile race. | The cohort overall had low to moderate general physical activity levels with 80% achieving less than 10,000 steps a day.  21.9% reported they had pain at the start of the running event.  They thought that stretching (75.4%) and running footwear (68%) were the most effective for injury prevention with less than a third reporting they thought resistance or balance training to be effective.  The authors suggest event organizers should provide injury prevention information to help runners prepare for an event. They suggest exercises professionals/ coaches should tailor activity programmes to optimize physical activity levels. |
| **Verhagen** | 2021 | qualitative | Recreational | The aim of this study was to find out recreational runners’ perspectives on injury management and prevention. | Four main themes were identified.  Motivation to participate in running - physical health benefits; social; performance; distraction from daily life.  Onset and care for complaints - injuries are generally caused by overloading; not resting and fatigue; a complaint or small pain is not an injury, but normal for runners; management of injury was through training modification; motivations to continue running with pain are influenced by performance goals, continuing everyday life activities, and being part of a running group/ not letting anyone down.  Definition and care of injury - injury was seen as something preventing activity; running experience influenced how they dealt with their injury; variability on how an injury was managed went from deal with it themselves or see a professional.  Self-regulation/ preventative behaviours - this was driven by the runner’s own experience, online information, or peer influences. Interestingly runners in this cohort were unable to identify what they consciously did for injury prevention other than change shoes regularly, use a training schedule, and do core exercises. However, they do look towards trainers as peers, and physiotherapists for expert advice on this. |
| **Zhao** | 2022 | Questionnaire | Recreational | The questionnaire investigated the use of sports protective equipment in runners. | excessive training loads, lack of preparation activities such as stretching and movement errors were thought to be the main factors causing injury. use of foam rollers and use of protective equipment such ankle support, knee pads and elbow pads were thought to help prevent injury |
