# Supplementary material for: Running-Centred Injury Prevention Support: A Scoping Review on Current Injury Risk Reduction Practices for Runners
Source: Transl Sports Med. 2025 Feb 25;2025:3007544. doi: 10.1155/tsm2/3007544 (PMC11986186; doi:10.1155/tsm2/3007544)
Supplement: Supporting Information 3 — Supporting file 3: Quality assessment RCTs. [file 3007544.f3.docx]

**Supplementary file 3: Quality assessment, PEDro Scale Scoring for Group 1 studies**

| **Author**  **Year** | **PEDro Scale** | | | | | | | | | | |
| --- | --- | --- | --- | --- | --- | --- | --- | --- | --- | --- | --- |
|  | **1** | **2** | **3** | **4** | **5** | **6** | **7** | **8** | **9** | **10** | **11** |
| Adriaensens  2014 [21] | ✓ | ✓ | ✓ | ✓ | ✓ | ✓ | ✓ | < 85% participants at study completion | ✓ | ✓ | ✓ |
| Baltich  2016 [51] | ✓ | ✓ | ✓ | ✓ | ✓ | Not specified | Not specified | < 85% participants at study completion | ✓ | ✓ | ✓ |
| Bertelsen  2018 [36] | ✓ | ✓ | ✓ | ✓ | Not blinded | Not blinded | Not blinded | < 85% participants at study completion | ✓ | ✓ | ✓ |
| Bredeweg  2012 [37] | ✓ | ✓ | ✓ | ✓ | Not specified | Not specified | Not specified | ✓ | ✓ | ✓ | ✓ |
| Buist  2008 [38] | ✓ | ✓ | ✓ | ✓ | Not specified | Not specified | Not specified | ✓ | ✓ | ✓ | ✓ |
| Chan  2018 [54] | ✓ | ✓ | ✓ | ✓ | Not specified | Not specified | Not specified | ✓ | ✓ | ✓ | ✓ |
| Cloostermann  2020 [22] | ✓ | ✓ | ✓ | ✓ | ✓ | ✓ | ✓ | ✓ | ✓ | ✓ | ✓ |
| Desai  2022 [52] | ✓ | No information on randomization | No information concealment | ✓ | Not specified | Not specified | Not specified | ✓ | ✓ | ✓ | ✓ |
| Dubois  2015 [29] | ✓ | ✓ | ✓ | ✓ | ✓ | ✓ | ✓ | ✓ | ✓ | ✓ | ✓ |
| Fokkema  2019 [23] | ✓ | ✓ | ✓ | ✓ | ✓ | ✓ | ✓ | < 85% participants at study completion | ✓ | ✓ | ✓ |
| Fuller  2017 [30] | ✓ | ✓ | ✓ | ✓ | Not blinded | Not blinded | Not blinded | ✓ | ✓ | ✓ | ✓ |
| Halvarsson 2019 [45] | ✓ | ✓ | ✓ | ✓ | Not blinded | Not blinded | ✓ | ✓ | ✓ | ✓ | ✓ |
| Hespanhol Junior 2018 [24] | ✓ | ✓ | ✓ | ✓ | ✓ | ✓ | ✓ | ✓ | ✓ | ✓ | ✓ |
| Hollman  2019 [25] | ✓ | ✓ | ✓ | ✓ | Not blinded | ✓ | ✓ | < 85% participants at study completion | ✓ | ✓ | ✓ |
| Jacobsson  2023 [26] | ✓ | ✓ | ✓ | ✓ | ✓ | ✓ | ✓ | < 85% participants at study completion | ✓ | ✓ | ✓ |
| Letafatkar  2019 [47] | ✓ | ✓ | Method of randomization not specified | ✓ | Not specified | Not specified | Not specified | ✓ | ✓ | ✓ | ✓ |
| Letafarkar  2020 [46] | ✓ | ✓ | Method of randomization not specified | ✓ | Not specified | Not specified | Not specified | ✓ | ✓ | ✓ | ✓ |
| Lundstrom  2019 [39] | ✓ | 2 intervention groups were randomly selected but the control group was not randomly selected from the same group of participants | No method of concealment | ✓ | Not specified | Not specified | Not specified | ✓ | ✓ | ✓ | ✓ |
| Malisoux  2016 [31] | ✓ | ✓ | ✓ | ✓ | ✓ | ✓ | ✓ | ✓ | ✓ | ✓ | ✓ |
| Malisoux  2016 [32] | ✓ | ✓ | ✓ | ✓ | ✓ | ✓ | ✓ | ✓ | ✓ | ✓ | ✓ |
| Malisoux  2019 [33] | ✓ | ✓ | ✓ | ✓ | ✓ | Not specified | Not specified | ✓ | ✓ | ✓ | ✓ |
| Mendez-Rebolledo 2012 [48] | ✓ | ✓ | No method of concealment | ✓ | Not specified | Unclear | Unclear | ✓ | ✓ | ✓ | ✓ |
| Ramskov  2018 [40] | ✓ | ✓ | ✓ | ✓ | ✓ | ✓ | ✓ | < 85% participants at study completion | ✓ | ✓ | ✓ |
| Suda  2022 [49] | ✓ | ✓ | ✓ | ✓ | ✓ | ✓ | ✓ | ✓ | ✓ | ✓ | ✓ |
| Taddei  2020 [50] | ✓ | ✓ | ✓ | ✓ | ✓ | ✓ | ✓ | ✓ | ✓ | ✓ | ✓ |
| Thiesen  2014 [34] | ✓ | ✓ | No method of concealment | ✓ | Not specified | Not specified | Not specified | ✓ | ✓ | ✓ | ✓ |
| Toresdahl  2020 [53] | ✓ | ✓ | No method of concealment | ✓ | Not specified | Not specified | Not specified | ✓ | ✓ | ✓ | ✓ |
| Van der Does 2023 [27] | ✓ | ✓ | ✓ | ✓ | Not blinded | Not blinded | Not blinded | ✓ | ✓ | ✓ | ✓ |
| Van Hooren 2024 [55] | ✓ | ✓ | ✓ | ✓ | Not specified | Not specified | Not blinded | < 85% participants at study completion | ✓ | ✓ | ✓ |
| Van Iperen 2022 [28] | ✓ | ✓ | No method of concealment | ✓ | Not specified | Not specified | Not specified | < 85% participants at study completion | ✓ | ✓ | ✓ |
| Willems  2021 [35] | ✓ | ✓ | ✓ | ✓ | ✓ | ✓ | ✓ | ✓ | ✓ | ✓ | ✓ |

PEDro scale:

1. eligibility criteria were specified

2. subjects were randomly allocated to groups (in a crossover study, subjects were randomly allocated an order in which treatments were received)

3. allocation was concealed

4. the groups were similar at baseline regarding the most important prognostic indicators

5. there was blinding of all subjects

6. there was blinding of all therapists who administered the therapy

7. there was blinding of all assessors who measured at least one key outcome

8. measures of at least one key outcome were obtained from more than 85% of the subjects initially allocated to groups

9. all subjects for whom outcome measures were available received the treatment or control condition as allocated or, where this was not the case, data for at least one key outcome was analysed by “intention to treat”

10. the results of between-group statistical comparisons are reported for at least one key outcome

11. the study provides both point measures and measures of variability for at least one key outcome
